# Self-esteem in adolescents with obesity: a scoping review^1^

**DOI:** 10.1590/1518-8345.7936.4888

**Published:** 2026-08-10

**Authors:** Chrisllayne Oliveira da Silva, Nanielle Silva Barbosa, Ana Larissa Gomes Machado, Ítalo Arão Pereira Ribeiro, Ana Maria Ribeiro dos Santos, Márcia Astrês Fernandes

**Affiliations:** 1 Universidade Federal do Piauí, Teresina, PI, Brazil.; 2 Scholarship holder at the Coordenação de Aperfeiçoamento de Pessoal de Nível Superior (CAPES), Brazil.; 3 Universidade Federal de Mato Grosso do Sul, Coxim, MS, Brazil.

**Keywords:** Adolescent, Self Concept, Obesity, Psychological Well-Being, Nursing, Review.

## Abstract

**Objective:**

to map the scientific evidence regarding the assessment, perception, and interventions aimed at self-esteem in adolescents with obesity.

**Method:**

a scoping review, whose protocol was registered on the Open Science Framework platform with the identifier 10.17605/OSF.IO/C6W72. Primary and secondary studies, available in full, without temporal or language restrictions, from eight databases and grey literature were included. Letters to the editor, studies in the protocol phase, or those without results were excluded.

**Results:**

39 studies were selected for analysis. The synthesis of evidence in the investigated adolescents with obesity indicated low self-esteem, which was associated with female sex, body shame, exposure to peer victimization, depressive symptoms, and poor school performance. Interventions with potential results in improving self-esteem were related to weight reduction, such as sports practices, educational programs, and family support.

**Conclusion:**

there is a complex relationship between obesity in adolescents and low self-esteem, making it essential to establish good eating habits and regular physical activity, in addition to emotional support for adolescents considering the biopsychosocial dimensions.

## Highlights

**(1)** Adolescents with obesity have lower self-esteem. **(2)** Low self-esteem affects physical and mental well-being and social relationships. **(3)** Family support is one of the factors that influence the formation of self-esteem. **(4)** Good eating habits and regular physical activity promote self-esteem.

## Introduction

Adolescence is generally considered a transitional phase, although the individual is not seen as a child, they are not yet considered an adult. It is precisely during this period that human development occurs rapidly, whether in physical, cognitive, social, sexual and emotional aspects^1^. These intense changes can contribute to situations that pose health risks to this adolescent and to the development of Non-communicable Chronic Diseases (NCDs) such as obesity, which is the excessive or abnormal accumulation of body fat^1^ ^-^ ^3^.

Adolescents with obesity constantly deal with external factors, such as difficulty in acceptance and inclusion in groups, social stigma, bullying, which can result in low levels of self-esteem, leading to a vulnerability to eating disorders, anxiety, depression and social isolation^4^ ^-^ ^5^.

In view of the above, the self-esteem of adolescents should be considered a relevant variable to assess the health of these individuals, given that it is linked to the construction of identity and adaptation to the environment, functioning as one of the regulators of well-being^6^ ^-^ ^7^.

In a nationwide survey of Brazilian students aged 12 to 17 years old, three out of ten students are dissatisfied with their body image, while adolescents with obesity are prone to low self-esteem and neglect of self-perception associated with excess weight and risks of comorbidities^2^.

In this sense, self-esteem, defined as a self-assessment of oneself, one’s own attitudes and relationship with others, influences how the individual reacts to internal and external changes, in addition to attributing favorable or unfavorable values and feelings that, when negative, impact the adolescent’s health. On the other hand, at satisfactory levels, they can influence the adoption of health-promoting attitudes and protection against harmful behaviors^6^ ^,^ ^8^ ^-^ ^9^.

Based on a preliminary literature search, two studies were identified regarding the relationship between obesity and self-esteem: a scoping review aimed at mapping existing instruments to measure self-esteem in children and adolescents, and a systematic review that aimed to assess whether multicomponent interventions would have an impact on adolescents’ self-esteem^10^ ^-^ ^11^. Thus, this study differs from others by addressing aspects related to the perception and concepts of adolescents with obesity regarding self-esteem and how this impacts daily life, in addition to understanding the relationship between obesity and self-esteem as a factor of illness or protection.

Despite the significant number of studies on obesity in adolescence in different databases, there is still a gap regarding self-esteem, which is an important factor in personal development and maintenance of health conditions.

Therefore, understanding the perception of adolescents with obesity can provide relevant evidence about the influences on self-image, considering that obesity can be a risk factor for a negative self-concept, and can be an aggravating factor for low self-esteem. Furthermore, the information obtained can contribute to the development of future research that can guide interventions with an emphasis on the complexities and mitigation of the stigma associated with obesity and its impacts on mental health.

This study aimed to map the scientific evidence regarding the assessment, perception, and interventions aimed at self-esteem in adolescents with obesity.

## Method

### Study design

This literature review, a scoping review, was conducted in accordance with the steps recommended by the JBI: title and review questions, inclusion criteria, search strategy, screening and selection of evidence, data extraction and analysis, and presentation of results; and reported using the guidelines of the Preferred Reporting Items for Systematic Reviews and Meta-Analyses extension for Scoping Review (PRISMA-ScR)^12^ ^-^ ^13^.

The review protocol was registered in the Open Science Framework (OSF) with DOI identifier 10.17605/OSF.IO/C6W72. Prior to developing the protocol, the authors checked the literature for other reviews with similar themes and objectives to this review. Two reviews were found, but they were directed to self-esteem assessment instruments and specific interventions, differing from the objectives of the present study^10^ ^-^ ^11^.

### Setting

The databases consulted were: Latin American and Caribbean Literature in Health Sciences (LILACS), Nursing Database (BDENF), Spanish Bibliographic Index in Health Sciences (IBECS), Psychology Index (INDEXPSI), via the Virtual Health Library (VHL), Medical Literature Analysis and Retrieval System Online (MEDLINE) via PubMed, Web of Science, Scopus, and APA PsycInfo^®^.

The grey literature was retrieved from sources such as the Brazilian Digital Library of Theses and Dissertations (BDTD), the Catalog of Theses and Dissertations (CTD) of the Coordination for the Improvement of Higher Education Personnel (CAPES), and Google Scholar (in the first 10 pages).

### Period

The research was conducted between December 2024 and January 2025.

### Population

The acronym PCC was used, with the population of adolescents, defined as individuals aged between 10 and 19 years, according to the World Health Organization (WHO) definition. The concept adopted was self-esteem, and the context of interest was obesity. The review question was: what evidence is available regarding the assessment, perception, and interventions aimed at self-esteem in adolescents with obesity? 

### Selection criteria

Primary and secondary studies on self-esteem in adolescents with obesity were included, both studies that considered these two variables as primary factors and those that analyzed different variables but verified self-esteem in adolescents with the comorbidity. All primary and secondary studies were available in full, without temporal or language restrictions. Letters to the editor, studies in the protocol phase, or those without results were excluded. 

### Sample definition

An initial literature search on the topic of interest was conducted with the assistance of a librarian. From the retrieved studies, an analysis of the terms contained in the title, abstract, text, and descriptors/keywords was performed in order to select them from the Health Sciences Descriptors (DeCS) and Medical Subject Headings (MeSH). These terms were combined to ensure the greatest possible return of studies, structuring search strategies specific to each database, as shown in Figure 1. The strategies were tested independently by each of the two reviewers and the librarian to ensure the same result in all searches.

**Figure 1:** Search strategy in databases and grey literature. Teresina, PI, Brazil, 2024

Database	Search strategie
LILACS*/BDENF^†^/IBECS^‡^/INDEXPSI^§^ (VHL^ǁ^)	((mh:(adolescente )) OR (adolescentes) AND (adolescência)) AND ((mh:(autoimagem)) OR (autoestima)) AND ((mh:(obesidade)) OR (obesidade)) AND db:(“LILACS*” OR “IBECS^‡^” OR “BDENF^†^” OR “INDEXPSI^§^”) AND instance:”regional”
MEDLINE^¶^ (PubMed)	(((“self concept”[MeSH Terms]) OR (“self esteem”[All Fields])) AND (((“adolescent”[MeSH Terms]) OR (“adolescents”[All Fields])) OR (“adolescence”[All Fields]))) AND ((Obesity[MeSH Terms]) OR (“obesity”[All Fields]))
Web of Science	((ALL=(Adolescent) OR ALL=(Adolescents) OR ALL=(Adolescence)) AND ALL=(Obesity)) AND (ALL=(‘’Self Concept’’) OR ALL=(‘’Self Esteem’’))
Scopus	( ( TITLE-ABS-KEY ( adolescent ) OR TITLE-ABS-KEY ( adolescents ) OR TITLE-ABS-KEY ( adolescence ) ) ) AND ( TITLE-ABS-KEY ( obesity ) ) AND ( ( TITLE-ABS-KEY ( ‘’self concept’’ ) OR TITLE-ABS-KEY ( self esteem ) ) )
APA** PsycInfo^®^	((Any Field: (Adolescent)) OR (Any Field: (Adolescents)) OR (Any Field: (Adolescence))) AND ((Any Field: (Self concept)) OR (Any Field: (Self Esteem))) AND ((Any Field: (Obesity)))
**Grey literature**	**Strategy**
BDTD^††^	(Adolescentes OR adolescente OR adolescência) AND (Autoimagem OR Autoestima) AND (Obesidade)
CTD-CAPES^‡‡^	(Adolescentes OR adolescente OR adolescência) AND (Autoimagem OR Autoestima) AND (Obesidade)
Google Scholar	Adolescents AND ‘’Self esteem’’ AND Obesity

*LILACS = Latin American and Caribbean Literature in Health Sciences; ^†^BDENF = Nursing Database; ^‡^IBECS = Spanish Bibliographic Index in Health Sciences; ^§^INDEXPSI = Psychology Index; ^ǁ^VHL = Virtual Health Library; ^¶^MEDLINE = Medical Literature Analysis and Retrieval System Online*;* **APA = American Psychological Association; ^††^BDTD = Brazilian Digital Library of Theses and Dissertations; ^‡‡^CTD-CAPES = Catalog of Theses and Dissertations from the Coordination for the Improvement of Higher Education Personnel

### Data collection

The search, selection, and analysis were conducted independently, blindly, by two reviewers, authors of the study, one with a Master’s degree in Health Sciences and knowledge in the area of Child and Adolescent Health, and the other with a Master’s degree in Nursing and knowledge in the area of Mental Health, both with experience in the preparation and publication of reviews. Disagreements regarding the inclusion or exclusion of a specific text, after discussion between the first two reviewers, were resolved by a third reviewer, a nurse with a PhD in Nursing and knowledge in the area of Public Health.

The identified citations were imported into the Rayyan^®^ reference manager, developed by the Qatar Computing Research Institute (QCRI), for duplicate removal, selection, and screening.

The first selection phase involved reading titles and abstracts. Studies that met the inclusion criteria were analyzed in a second phase, via reading the manuscripts in full.

The reasons for excluding studies were recorded and reported in a flowchart^14^.

### Study variables

The variables extracted from the studies were: authorship, country of origin, language, year of publication, objective, type of study, sample, data collection location, instrument used to assess self-esteem, main results, and level of evidence^15^.

### Data collection instrument

The information was mapped according to JBI recommendations for characterizing sources of evidence, recorded, and organized in a synoptic table format created in Microsoft Office Excel 2024.

### Data processing and analysis 

The data analysis was performed using descriptive, quantitative, and qualitative methods. The results were summarized in a narrative format and through figures.

### Ethical aspects

The study used secondary data, therefore, it was not reviewed by the Research Ethics Committee (REC).

## Results

**Figure 2:**
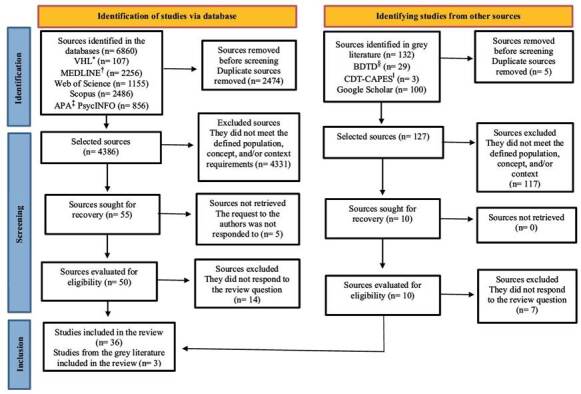
Study selection flowchart. Teresina, PI, Brazil, 2024

A total of 6860 studies were identified in the databases, of which 2472 were excluded due to duplicates and 4386 were proceeded to title and abstract reading. After the first reading, 50 were selected for full-text reading, of which 36 were included in the final sample. From the grey literature, three studies were included. Thus, 39 studies comprised the final sample of the review, as shown in Figure 2.

The description of the studies according to country of origin, year of publication, language, type of study, level of evidence, sample, location, and instrument for assessing self-esteem can be found in Figure 3.

**Figure 4:**
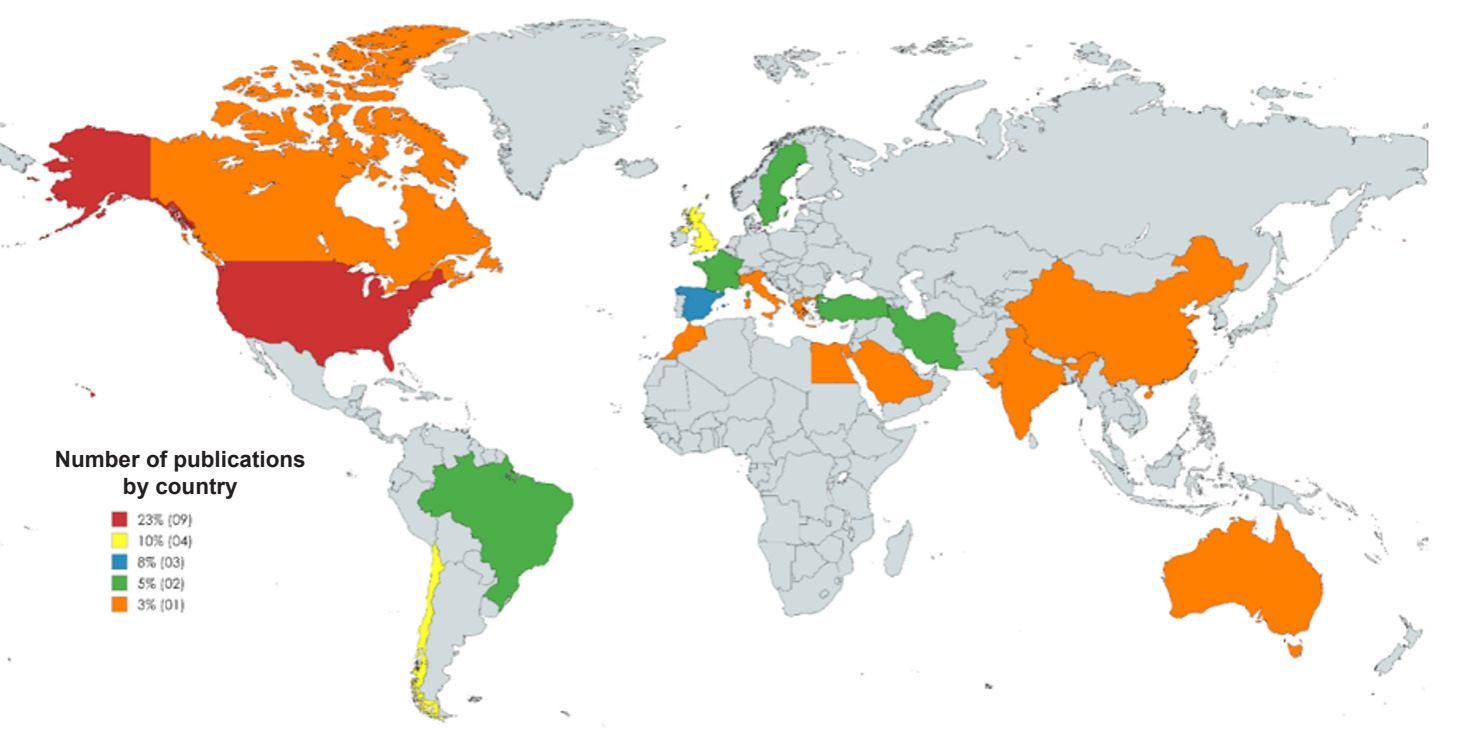
Country of origin and number of corresponding publications. Teresina, PI, Brazil, 2024

Regarding the country of origin of the studies, nine were conducted in the United States of America^23^ ^,^ ^25^ ^,^ ^34^ ^,^ ^41^ ^,^ ^43^ ^-^ ^46^ ^,^ ^53^, four in Chile^17^ ^,^ ^27^ ^-^ ^28^ ^,^ ^31^ and the United Kingdom^20^ ^-^ ^21^ ^,^ ^37^ ^,^ ^50^, three in Spain^29^ ^,^ ^39^ ^-^ ^40^, two in Brazil^16^ ^,^ ^33^, France^48^ ^,^ ^52^, Iran^26^ ^,^ ^42^, Sweden^47^ ^,^ ^51^ and Turkey^19^ ^,^ ^49^ and one in Saudi Arabia^35^, Australia^11^, Canada^38^, China^22^, Egypt^30^, India^32^, Italy^36^, Greece^24^ and Morocco^18^. A representative map of the countries of origin of the publications is presented in Figure 4.

*VHL = Virtual Health Library; ^†^MEDLINE = Medical Literature Analysis and Retrieval System Online; ^‡^APA = American Psychological Association; ^§^BDTD = Brazilian Digital Library of Theses and Dissertations; ^ǁ^CTD-CAPES = Catalog of Theses and Dissertations from the Coordination for the Improvement of Higher Education Personnel

**Figure 3:** Description of the selected studies regarding country of origin, year of publication, language, type of study, level of evidence, sample, location, and instrument for assessing self-esteem. Teresina, PI, Brazil, 2024

Order	Country of origin/year of publication/language	Method
01^16^	Brazil/2024/English	Study Type/Level of Evidence: Randomized controlled clinical trial/II Sample: 40 adolescents received the intervention and 13 participated in the control group, aged between 10 and 17 years old. Location: Study center. Instrument: Rosenberg Self-Esteem Scale.
02^17^	Chile/2024/Spanish	Study Type/Level of Evidence: Cross-sectional/IV Sample: 397 adolescents aged 10 to 19 years old. Location: Schools. Instrument: Rosenberg Self-Esteem Scale.
03^18^	Morocco/2024/English	Study Type/Level of Evidence: Cross-sectional/IV Sample: 410 adolescents aged 12 to 17 years old. Location: Schools. Instrument: Rosenberg Self-Esteem Scale.
04^19^	Turkey/2023/English	Study Type/Level of Evidence: Case-control/IV Sample: 50 children diagnosed with obesity and 48 participants in the control group, aged between 11 and 17 years old. Location: University. Instrument: Rosenberg Self-Esteem Scale.
05^20^	United Kingdom/2023/English	Study Type/Level of Evidence: Prospective cohort/IV Sample: 18,818 participants aged 11 to 17 years old. Location: Not specified. Instrument: Rosenberg Self-Esteem Scale.
06^21^	United Kingdom/2022/English	Study Type/Level of Evidence: Cross-sectional/IV Sample: 9046 adolescents aged 11 to 14 years old. Location: residences. Instrument: Rosenberg Self-Esteem Scale.
07^22^	China/2021/English	Study Type/Level of Evidence: Case-control/IV Sample: 128 students with obesity and 128 with normal weight. Location: School. Instrument: Body Stereotype Scale for Adolescents and Adults.
08^23^	United States of America/2021/English	Study Type/Level of Evidence: Cross-sectional/IV Sample: 6,748 adolescents. Location: Schools. Instrument: Modified version of the Rosenberg Self-Esteem Inventory..
09^24^	Greece/2020/English	Study Type/Level of Evidence: Cross-sectional/IV Sample: 528 adolescents aged 10 to 12 years. Location: School. Instrument: Self-Perception Profile for Children.
10^25^	United States of America/2020/English	Study Type/Level of Evidence: Cross-sectional/IV Sample: 160 adolescents aged 14 to 16 years old. Location: Community centers. Instrument: Rosenberg Self-Esteem Scale.
11^26^	Iran/2020/English	Study Type/Level of Evidence: Cross-sectional/IV Sample: 173 adolescents aged 12 to 16 years old. Location: Study center. Instrument: Rosenberg Self-Esteem Scale.
12^27^	Chile/2019/Spanish	Study Type/Level of Evidence: Cross-sectional/IV Sample: 419 adolescents aged 14 to 17 years old. Location: School. Instrument: Coopersmith Self-Esteem Inventory.
13^28^	Egypt/2019/English	Study Type/Level of Evidence: Cross-sectional/IV Sample: 533 adolescents aged 10 to 19 years old. Location: School. Instrument: Rosenberg Self-Esteem Scale.
14^29^	Chile/2018/English	Study Type/Level of Evidence: Cross-sectional/IV Sample: 712 schoolchildren aged 10 to 13 years old. Location: school. Instrument: SST-student^*^.
15^30^	Spain/2018/Spanish	Study Type/Level of Evidence: Cross-sectional/IV Sample: 364 schoolchildren aged 11 to 13 years old. Location: Public educational centers. Instrument: SST-student^*^.
16^31^	Chile/2018/English	Study Type/Level of Evidence: Cross-sectional/IV Sample: 656 adolescents aged 11 to 14 years old. Location: School. Instrument: SST-student^*^.
17^32^	India/2017/English	Study Type/Level of Evidence: Cross-sectional/IV Sample: 150 adolescents with a mean age of 14.5 years old. Location: School. Instrument: Rosenberg Self-Esteem Scale.
18^33^	Brazil/2017/Portuguese	Study Type/Level of Evidence: Cross-sectional/IV Sample: 418 adolescents aged 14 to 18 years old. Location: school. Instrument: Rosenberg Self-Esteem Scale.
19^34^	United States of America/2017/English	Study Type/Level of Evidence: prospective cohort/IV Sample: 139 adolescents aged 13 to 18 years old. Location: hospital. Instrument: Self-Perception Profile for Adolescents.
20^11^	Australia/2017/English	Study Type/Level of Evidence: Systematic review and meta-analysis/I Sample: Thirteen studies with 1,157 overweight or obese adolescents, aged 10 to 19 years old. Location: School and homes. Instrument: Rosenberg Self-Esteem Scale; Harter Self-Perception Profile for Children; Marsh Global Self-Esteem Scale.
21^35^	Saudi Arabia/2016/Arab	Study Type/Level of Evidence: retrospective cohort/IV Sample: 257 adolescents with a mean age of 18 years old. Location: school. Instrument: Rosenberg Self-Esteem Scale.
22^36^	Italy/2016/English	Study Type/Level of Evidence: Case-control/IV Sample: 111 students with obesity and 111 with normal weight, aged 13 to 19 years old. Location: School. Instrument: Rosenberg Self-Esteem Scale.
23^37^	United Kingdom/2016/English	Study Type/Level of Evidence: Randomized controlled clinical trial/II Sample: 303 young people aged 11 to 17 years old. Location: residences. Instrument: Self-Perception Profile for Children.
24^38^	Canada/2015/English	Study Type/Level of Evidence: Randomized controlled clinical trial/II Sample: 304 participants aged 14 to 18 years old. Location: Community facility. Instrument: Physical Self-Perception Profile for Children; Harter Global Self-Esteem Subscale.
25^39^	Spain/2015/Spanish	Study Type/Level of Evidence: Cross-sectional/IV Sample: 89 adolescents aged 12 to 16 years old. Location: Hospital. Instrument: Child Health and Disease Profile.
26^40^	Spain/2015/Spanish	Study Type/Level of Evidence: Cross-sectional/IV Sample: 296 children aged 12 to 14 years old. Location: School. Instrument: Rosenberg Self-Esteem Scale.
27^41^	United States of America/2014/English	Study Type/Level of evidence: case-control/IV Sample: 1419 adolescent students. Location: school. Instrument: General Self-Esteem Scale.
28^42^	Iran/2014/English	Study Type/Level of Evidence: Cross-sectional/IV Sample: 8,510 adolescents aged 15 to 17 years old. Location: Schools. Instrument: Body-Self Multidimensional Relationships Questionnaire; Coopersmith Self-Esteem Inventory.
29^43^	United States of America/2012/English	Study Type/Level of Evidence: Case-control/IV Sample: 101 adolescents aged 14 to 18 years old. Location: School. Instrument: Rosenberg Self-Esteem Scale.
30^44^	United States of America/2012/English	Study Type/Level of Evidence: Cross-sectional/IV Sample: 119 adolescents aged 11 to 18 years old. Location: Hospital. Instrument: Coopersmith Self-Esteem Inventory.
31^45^	United States of America/2011/English	Study Type/Level of Evidence: prospective cohort/IV Sample: 806 adolescents. Location: school. Instrument: Rosenberg Self-Esteem Scale.
32^46^	United States of America/2010/English	Study Type/Level of Evidence: prospective cohort/IV Sample: 4458 adolescents aged 12 to 16 years old. Location: remote. Instrument: Harter Self-Esteem Profile for Children.
33^47^	Sweden/2010/English	Study Type/Level of Evidence: prospective cohort/IV Sample: a cohort from the 2000s with 191 adolescents and a cohort from 2008 with 170 adolescents. Setting: school. Instrument: Physical Self-Perception Profile for Children and Adolescents.
34^48^	France/2010/French	Study Type/Level of Evidence: Randomized controlled clinical trial/II Sample: 16 adolescents with a mean age of 14.9 years old. Location: Hospital. Instrument: Physical Self-Awareness Inventory.
35^49^	Turkey/2004/English	Study Type/Level of Evidence: Case-control/IV Sample: 90 adolescents aged 12 to 16 years old. Location: School. Instrument: Rosenberg Self-Esteem Scale.
36^50^	United Kingdom/2023/English	Study Type/Level of Evidence: Randomized controlled clinical trial/II Sample: 57 obese adolescents and 38 adolescents with normal weight. Location: residential camp. Instrument: Self-Perception Profile for Children.
37^51^	Sweden/1999/English	Study Type/Level of Evidence: Case-control/IV Sample: 58 adolescents with obesity and 58 with normal weight, aged between 14 and 18 years old. Location: School. Instrument: I Think, I Am Questionnaire.
38^52^	France/1995/English	Study Type/Level of Evidence: Cross-sectional/IV Sample: 93 adolescents aged 13 to 19 years old. Location: Hospital. Instrument: Coopersmith Self-Esteem Inventory.
39^53^	United States of America/1988/English	Study Type/Level of Evidence: Cross-sectional/IV Sample: 550 girls aged 14 to 16 years old. Location: School. Instrument: Rosenberg Self-Esteem Scale.

*SST-student = School Self-Esteem Test

Regarding the year of publication, four were published in 2017^11^ ^,^ ^32^ ^-^ ^34^, three in 2024^16^ ^-^ ^18^, 2020^24^ ^-^ ^26^, 2018^29^ ^-^ ^31^, 2016^35^ ^-^ ^37^, 2015^38^ ^-^ ^40^, and 2010^46^ ^-^ ^48^, two in 2023^19^ ^-^ ^20^, 2021^22^ ^-^ ^23^, 2019^27^ ^-^ ^28^, 2014^31^ ^,^ ^42^ ^)^ e 2012^43^ ^-^ ^44^, one in 2022^21^, 2011^45^, 2004^49^, 2003^50^, 1999^51^, 1995^52^ and 1988^53^. Regarding the language, most studies were published in English^11^ ^,^ ^16^ ^,^ ^18^ ^-^ ^26^ ^,^ ^28^ ^-^ ^29^ ^,^ ^31^ ^-^ ^32^ ^,^ ^34^ ^,^ ^36^ ^-^ ^38^ ^,^ ^41^ ^-^ ^47^ ^,^ ^49^ ^-^ ^53^, followed by Spanish^17^ ^,^ ^27^ ^,^ ^20^ ^,^ ^39^ ^-^ ^40^, Portuguese^33^, Arabic^35^ and French^48^.

The types of studies developed were cross-sectional^17^ ^-^ ^18^ ^,^ ^21^ ^,^ ^23^ ^-^ ^33^ ^,^ ^39^ ^-^ ^40^ ^,^ ^42^ ^,^ ^44^ ^,^ ^52^ ^-^ ^53^, case-control^19^ ^,^ ^22^ ^,^ ^36^ ^,^ ^41^ ^,^ ^43^ ^,^ ^50^ ^,^ ^52^, cohort^20^ ^,^ ^34^ ^,^ ^36^ ^,^ ^46^ ^-^ ^48^, randomized controlled clinical trials^16^ ^,^ ^38^ ^-^ ^39^ ^,^ ^49^ ^,^ ^51^ and a systematic review^11^, with sample sizes ranging from 16^48^ to 18818^20^ adolescents. The Level of Evidence was classified, in such a way that studies of level VI^17^ ^-^ ^18^ ^,^ ^21^ ^,^ ^23^ ^-^ ^33^ ^,^ ^39^ ^-^ ^40^ ^,^ ^42^ ^,^ ^44^ ^,^ ^52^ ^-^ ^53^, IV^19^ ^-^ ^20^ ^,^ ^22^ ^,^ ^34^ ^,^ ^36^ ^,^ ^41^ ^,^ ^43^ ^,^ ^46^ ^-^ ^48^ ^,^ ^50^ ^,^ ^52^, II^16^ ^,^ ^38^ ^-^ ^39^ ^,^ ^49^ ^,^ ^51^ and I^11^ were identified. The studies were carried out in different settings such as camp^50^, community centers^25^, study center^26^, public educational centers^29^, schools^11^ ^,^ ^17^ ^-^ ^18^ ^,^ ^22^ ^-^ ^24^ ^,^ ^27^ ^-^ ^28^ ^,^ ^30^ ^-^ ^33^ ^,^ ^35^ ^-^ ^36^ ^,^ ^40^ ^-^ ^43^ ^,^ ^45^ ^,^ ^47^ ^,^ ^49^ ^,^ ^51^ ^,^ ^53^, hospitals^33^ ^,^ ^39^ ^,^ ^44^ ^,^ ^48^ ^,^ ^52^, community facilities^38^, remote^46^, residences^11^ ^,^ ^21^ ^,^ ^37^ and university^19^, and in one of them it was not specified^20^.

The instruments Global Self-Esteem of Marsh^11^, Rosenberg Self-Esteem Scale^11^ ^,^ ^16^ ^-^ ^21^ ^,^ ^25^ ^-^ ^26^ ^,^ ^28^ ^,^ ^30^ ^,^ ^32^ ^-^ ^33^ ^,^ ^36^ ^,^ ^40^ ^,^ ^41^ ^,^ ^45^ ^,^ ^49^ ^,^ ^53^ and modified version^23^, Body Stereotype Scale for Adolescents and Adults^22^, General Self-Esteem Scale^41^, Coopersmith Self-Esteem Inventory^27^ ^,^ ^42^ ^,^ ^44^ ^,^ ^52^, Physical Self-Inventory^48^, Harter Self-Esteem Profile for Children^46^, Harter Self-Perception Profile for Children^11^, Physical Self-Perception Profile for Children^38^, Physical Self-Perception Profile for Children and Adolescents^47^, Self-Perception Profile for Adolescents^34^, Self-Perception Profile for Children^24^ ^,^ ^37^ ^,^ ^50^, Child Health and Illness Profile^39^, I Think, I Am Questionnaire^51^, Multidimensional Relationships Questionnaire Body-Self^42^, Harter Global Self-Esteem Subscale^38^ and SST-student: school self-esteem test^29^ ^-^ ^31^ were used to assess self-esteem among participants.

Studies have identified that obesity and increased Body Mass Index (BMI) influence low self-esteem among adolescents, highlighting this prevalence among females^11^ ^,^ ^17^ ^-^ ^24^ ^,^ ^27^ ^-^ ^30^ ^,^ ^33^ ^,^ ^39^ ^-^ ^43^ ^,^ ^45^ ^-^ ^47^ ^,^ ^51^ ^-^ ^53^. It is pointed out that low self-esteem contributes to body shame^22^ ^,^ ^36^, one of the predictors for Eating Disorders, as well as being influenced by exposure to peer victimization^41^ ^,^ ^44^, reflecting in negative impacts on mental health and well-being^19^ ^-^ ^21^ ^,^ ^26^ ^,^ ^32^ ^,^ ^41^ ^,^ ^43^ ^,^ ^48^ ^-^ ^49^ and school performance^27^ ^,^ ^34^ ^,^ ^35^. The need for interventions to promote self-esteem in this population is highlighted, such as sports and physical activity, and the importance of family support^16^ ^,^ ^25^ ^-^ ^26^ ^,^ ^37^ ^-^ ^38^ ^,^ ^40^ ^,^ ^50^. The objectives and main results of the studies are described in Figure 5.

**Figure 5:** Description of the selected studies regarding their objectives and main results. Teresina, PI, Brazil, 2024

Order	Objectives	Main results
01^16^	To analyze the effects of 16 weeks of Muay Thai practice on body perception, self-esteem, and QoL* in overweight/obese adolescents.	An increase in self-esteem was observed among participants in the group that practiced Muay Thai.
02^17^	Relating body dissatisfaction to self-esteem, depression, and BMI^†^ in adolescents.	Of the sample, 17.1% were obese and 1.5% were severely obese. Low self-esteem was observed to be prevalent among females. Correlations were found between body dissatisfaction variables and BMI z-score^†^, depressive symptoms, and self-esteem.
03^18^	To assess levels of self-esteem, depression, and their association with obesity/overweight.	In the obesity category, girls had significantly lower self-esteem compared to boys. A strong association was found between depression and obesity/overweight and low self-esteem.
04^19^	To investigate the effects of obesity on anxiety, depression, self-esteem, and emotional regulation in children and adolescents.	Secondary analyses showed that children with obesity had lower self-esteem than those with symptoms of anxiety and depression. A correlation was also found between symptoms of depression, anxiety, and difficulties in emotional regulation in the obese group.
05^20^	To determine intervention pathways between mental health and BMI z-score symptoms^†^ throughout adolescence.	Evidence was found that the relationship between BMI^†^ and mental health was partially attributable to happiness with appearance and self-esteem. For both boys and girls, elevated emotional symptoms compared to low/moderate symptoms and an increase in BMI^†^ z-score at age 11 were associated with higher odds of unhappiness with appearance and low self-esteem at 14 years old.
06^21^	To investigate gender-specific characteristics associated with low sports participation among adolescents in the United Kingdom and to assess the tracking of gender-specific BMI^†^ and gender-specific associations between BMI^†^ and self-esteem based on different levels of adolescent sports participation.	Boys and girls in the lowest quartile of sports participation were more likely to be overweight/obese and have low self-esteem compared to adolescents in the highest quartile of sports participation.
07^22^	To explore how emotional intelligence, gender, and body size influence the relationship between body self-esteem and the risk of eating disorders among adolescents.	Gender and body size directly influenced body self-esteem and the risk of eating disorders. Furthermore, emotional intelligence acted as a moderator between body self-esteem and the risk of eating disorders.
08^23^	Expanding the literature by using a relative measure of body weight or a student’s BMI^†^ ranking within their peer group is relevant for examining the impact of body weight on youth outcomes.	Female students with a high BMI^†^ are more likely to experience decreased self-esteem and increased depressive symptoms.
09^24^	To investigate excess body weight and abdominal obesity in relation to self-perception, self-esteem, and anxiety.	Overweight and obese participants had lower scores in all domains of global self-esteem compared to children of normal weight.
10^25^	To examine the role of cultural (e.g., familism) and psychosocial (e.g., self-esteem) factors as predictors of weight-specific QoL* among obese Latino adolescents.	Family support and positive self-esteem may serve as protective factors associated with higher QoL* in adolescents with obesity, while self-deprecation may be a risk factor for lower QoL* associated with weight.
11^26^	To investigate the impact of communication and family function, and satisfaction with body image, on psychological well-being, considering the mediating role of self-esteem and depression.	The results showed that communication and family function directly affected the psychological well-being of adolescents. Furthermore, communication and family function, as well as satisfaction with body image, indirectly affected psychological well-being through self-esteem and depression.
12^27^	To determine the association between nutritional levels, physical fitness, self-esteem, and QoL* with academic performance in adolescents.	Schoolchildren with obesity presented lower HRQoL^‡^ than their peers with overweight and normal weight. Schoolchildren with overweight and obesity presented lower levels of self-esteem, which was associated with poor academic performance.
13^28^	To measure the prevalence of overweight and obesity and identify its relationship with self-esteem among school-aged adolescents.	Study results show that 71 (13.3%) adolescents are obese. Furthermore, a statistically significant association was identified between self-esteem and BMI^†^, with low self-esteem present in approximately three-quarters of overweight and obese adolescents.
14^29^	To determine if there is an association between body image dissatisfaction and anthropometric parameters, weight status, and self-esteem in children from public schools.	Body image dissatisfaction was greater among girls and children with obesity, who also exhibited lower self-esteem.
15^30^	To relate overweight and obesity status to levels of self-esteem and happiness in students from public educational centers.	School self-esteem was significantly higher in boys. Excess weight was associated with unhappiness in school-aged children.
16^31^	To evaluate the perception of obese schoolchildren regarding their participation in physical education classes and to determine their self-esteem in comparison with overweight and normal-weight groups.	Schoolchildren with obesity feel excluded from physical education classes and exhibit lower levels of self-esteem compared to schoolchildren of normal weight.
17^32^	To examine whether obese/overweight adolescent girls differ from normal-weight adolescent girls in terms of self-esteem.	Correlational analysis of the data revealed a negative relationship between BMI^†^ and self-esteem. The findings suggested a need to improve the self-esteem of adolescent girls, which generally decreases due to perceptions of body weight, as well as due to the social and psychological factors involved.
18^33^	To investigate self-esteem, body image, and depression in adolescents with different nutritional statuses.	Boys showed a higher prevalence of overweight and obesity, lower rates of depression and body dissatisfaction, and higher self-esteem than girls. Depression was negatively related to self-esteem, which was also negatively associated with body dissatisfaction.
19^34^	To examine the associations of peer victimization with internalizing symptoms, social competence, and academic performance in a clinical sample of adolescents with severe obesity, and whether self-esteem and social support affect these associations.	Self-esteem mediated the effects of victimization, being important for the prevention and intervention of both victimization and maladjustment in severe obesity among adolescents.
20^11^	To investigate the impact of multicomponent weight management interventions on self-esteem in overweight and obese adolescents.	Overweight and obese adolescents tend to have low self-esteem. The meta-analysis found no significant improvement in self-esteem, even with weight loss. However, the interventions with the greatest effects on self-esteem also showed the greatest effect on weight loss.
21^35^	Assessing the association between overweight/obesity and future overall academic performance among high school students in Saudi Arabia.	In a multiple logistic regression model adjusted for sociodemographic characteristics, study-related lifestyle, and self-esteem, overweight/obesity was associated with a decline in academic performance.
22^36^	To investigate dysfunctional eating behaviors and psychological variables typically associated with eating disorders, such as low self-esteem, perfectionism, shame, perceived parental care, and protectiveness in obese and normal-weight adolescents, and to examine how the main risk factors for eating disorders interact with each other, which explains vulnerability to eating psychopathology.	Self-esteem did not have a direct effect on the risk of eating disorders. Body shame had a stronger relationship with vulnerability to eating problems; however, low self-esteem had a direct influence on body shame, which in turn affects the risk of eating psychopathology.
23^37^	To assess perceived self-competence and low self-esteem during an intensive weight loss program in a large sample of obese young people and to relate them to initial weight, gender, and weight loss.	Participants who attended the summer camp experienced improvements in overall self-esteem, reducing low self-esteem from 35% to 16%.
24^38^	To determine the effects of aerobic training, resistance training, and combined training on mood, body image, and self-esteem in obese adolescents.	Based on the aerobic, resistance, and combined training interventions, all groups showed improvements in body image and physical self-perception. Resistance training showed the greatest increases in overall self-esteem.
25^39^	To assess the influence of weight status on the HRQoL^‡^ of adolescents.	Overweight/obese adolescents presented with worse HRQoL^‡^, lower resistance, lower capacity for physical activity, less family involvement, and greater peer influence. Self-esteem was lower among female participants.
26^40^	To analyze the influence of body composition on self-esteem in children aged 12 to 14 years old in the city of Jaén, Spain.	Statistically lower self-esteem values were observed in girls compared to boys. Higher BMI^†^ was also associated with lower self-esteem.
27^41^	To examine group differences related to provocations in aspects of psychological well-being, self-perceptions, and behaviors.	Studies have identified a correlation between teasing about weight and reduced psychological well-being. Adolescents who reported experiencing teasing exhibited lower self-esteem, more depressive symptoms, and perceived themselves as less physically capable compared to those who were not teased.
28^42^	To examine adolescents’ satisfaction with their body image in relation to their self-esteem.	Self-esteem was low among adolescents with obesity. Lower satisfaction with body image leads to low self-esteem.
29^43^	To compare the levels of stress, self-esteem, coping, social support, and depressive mood among minority adolescents with normal weight and those with overweight/obese.	Adolescents who were obese/overweight reported significantly lower self-esteem than those with normal weight. Low self-esteem was also a significant predictor of depressive mood in the overweight/obese group.
30^44^	To examine weight-based correlates of self-esteem and depression among 119 obese African American adolescents aged 11 to 18 years old seeking treatment.	The results indicated an association between teasing and decreased self-esteem and increased depression. Body satisfaction partially mediated the association between teasing and self-esteem.
31^45^	To test the hypothesis that body dissatisfaction mediates the association between obesity and impairment in emotional well-being at two points in the lives of a sample of the general population of male and female adolescents.	Obese participants tended to have lower self-esteem and higher levels of depressive mood than participants of normal weight. Furthermore, absolute levels of body dissatisfaction and depressive mood remained higher and self-esteem levels lower for girls, regardless of body weight.
32^46^	To examine characteristics associated with low self-esteem in a large national sample of young adolescents.	In the multivariate analysis, female sex, Hispanic race, overweight and obesity, sensation seeking, rebelliousness, and daily television viewing time were independently associated with low self-esteem.
33^47^	To explore secular trends in physical self-esteem between 2000 and 2008 by comparing cross-sectional cohorts of young adolescents.	There was significantly higher Global Self-Esteem in the 2008 cohort compared to the 2000 cohort. In the 2008 cohort, among the overweight/obese adolescent group, the only significant difference was lower perceived Physical Strength in girls and significantly lower Global Self-Esteem in boys.
34^48^	To analyze the relationships between anxiety levels, overall self-esteem, and perceived exertion during progressively increasing intensity physical exercise in a population of obese and non-obese adolescents.	Anxiety levels recorded before exercise are significantly correlated with overall self-esteem scores in the obese group.
35^49^	To explore the type and frequency of psychopathology in a clinical and non-clinical sample of obese adolescents and in a normal weight control group.	There was a higher proportion of psychopathology (depression, behavioral problems, low self-esteem) among clinically obese adolescents than among non-clinically obese adolescents. The findings provided evidence of a psychosocially at-risk population within a subgroup of obese adolescents.
36^50^	To investigate changes in body image, self-esteem, and concerns in obese adolescents attending a residential weight-loss camp.	Participants showed greater dissatisfaction with their body weight and self-esteem. Participation in the weight camp improved the adolescents’ psychological state and overall levels of self-esteem.
37^51^	To compare self-esteem, social history, social and academic competence, behavioral problems, and lifestyle of adolescents with obesity.	Obesity showed a non-significant correlation with self-rated self-esteem. Although this relationship was limited, obese participants demonstrated dissatisfaction with their physical appearance and athletic abilities, as expected. These findings indicate that, despite being overweight, adolescents’ self-esteem perception was not strongly influenced by obesity.
38^52^	To study eating and emotional disorders in adolescent girls with insulin-dependent Diabetes Mellitus.	Girls with obesity, with or without Diabetes Mellitus, presented lower scores on the total self-esteem scale.
39^53^	To investigate the self-esteem of adolescent girls in relation to their weight.	The results demonstrate that the self-esteem of adolescent girls is related to their weight. As the obesity rate increased, self-esteem decreased.

^*^ QoL = Quality of life; ^†^BMI = Body Mass Index; ^‡^HRQoL = Health-Related Quality of Life

## Discussion

Mapping the studies allowed us to identify how self-esteem is characterized among adolescents with obesity, its impacts, and possible interventions for improvement. Most studies indicate that the diagnosis of obesity among adolescents is related to lower self-esteem^11^ ^,^ ^17^ ^-^ ^19^ ^,^ ^21^ ^,^ ^24^ ^,^ ^27^ ^,^ ^31^ ^,^ ^41^ ^-^ ^43^ ^,^ ^51^. The relationship between self-esteem and BMI was examined in different investigations that revealed a negative and significant correlation, suggesting that as BMI increases, self-esteem decreases^20^ ^,^ ^30^ ^,^ ^32^. An inverse relationship can also be observed in the literature, indicating that having low self-esteem is one of the main factors that contribute to obesity and affect its treatment^19^ ^,^ ^54^.

Obesity reflects in lower self-esteem because it can negatively influence self-perception during adolescence, especially among females^18^ ^,^ ^22^ ^-^ ^23^ ^,^ ^28^ ^-^ ^29^ ^,^ ^33^ ^,^ ^39^ ^-^ ^40^ ^,^ ^45^ ^-^ ^46^ ^,^ ^52^ ^-^ ^53^. This difference between genders is influenced by sociocultural, family, and individual experience factors. The media, beauty standards considered acceptable, social expectations, and academic pressures can impact self-esteem in distinct ways, contributing to the formation of unique identities and self-perceptions between the genders^55^.

Low self-esteem is associated with life dissatisfaction, family problems, less perceived social support, negative emotional health and well-being, and mental disorders such as depression and anxiety, considered a predictor of higher suicide rates in this group^23^ ^,^ ^32^ ^,^ ^41^ ^,^ ^43^ ^,^ ^49^ ^,^ ^56^ ^-^ ^57^. Another point is that symptoms of anxiety and depression, common in obesity, contribute to decreased levels of self-esteem^19^ ^-^ ^20^ ^,^ ^45^. It is noteworthy that the influence of body image, social acceptance, and mood deterioration is greater in females, a fact that makes them more vulnerable to psychosocial disorders^58^ ^-^ ^59^.

Low self-esteem among adolescents with obesity can also be considered a risk factor for clinical manifestations of Eating Disorders, especially among women, as the pursuit of the body considered “ideal” can lead to the adoption of inappropriate eating behaviors^22^. Low self-esteem also directly influences body shame, which in turn is a risk predictor for eating psychopathologies^36^.

Prejudice against obesity and teasing about weight are common in the social environment^41^. There is evidence that being obese is identified as one of the least socially acceptable and possibly most stigmatizing conditions, especially in childhood and adolescence^60^. Self-esteem levels of children with obesity decrease with age and psychosocial difficulties become more pronounced as they grow and enter adolescence, as adolescents with obesity are more likely to suffer teasing from their peers, which can lead to social marginalization^24^ ^,^ ^61^.

Exposure to weight-related teasing, bullying, or victimization also contributes to the development of psychological and emotional problems, leading to poor school performance^27^ ^,^ ^34^ ^-^ ^35^. It has also been reported that school performance is influenced by excess weight or absenteeism among obese students due to the health problems they may face^24^. Different explanations have been suggested for the negative association between BMI and academic performance, and one of them includes the negative impact on self-esteem^35^.

Although this review highlights the negative relationship between obesity and self-esteem, the latter, when evaluated positively, is related to better well-being and mental health, positive self-perceptions, academic achievements, and persistence^31^. This reinforces the importance of preventing and controlling obesity in childhood, as well as counseling strategies to help control excess weight among adolescents^46^. Studies show that adolescents with better academic performance and participation in team sports have a lower risk of low self-esteem, highlighting an opportunity to encourage the development of school and community sports programs that contribute to the prevention of obesity and the promotion of better self-esteem^21^ ^,^ ^31^ ^,^ ^46^. Individuals with higher self-esteem also feel more motivated to engage in physical activity^18^ ^,^ ^21^ ^,^ ^62^.

Sports practices, such as Muay Thai, have been applied as an intervention to improve self-esteem among adolescents with obesity^16^. These practices contribute not only to physical and mental health, but also promote social skills^63^. A study that offered a Karate program lasting more than ten weeks to children and adolescents with epilepsy showed improvements in the self-esteem of the practitioners^64^. Another study analyzed self-esteem, self-confidence, and cognitive and somatic anxiety in adults who practice and do not practice Jiu-Jitsu, and observed that practitioners showed higher levels of self-esteem and self-confidence^65^.

Aerobic and resistance training, and the combination of both, demonstrated positive effects on the psychological health of obese adolescents. It was found that practice, at a frequency of at least six months, resulted in improvements in overall and physical self-esteem. Factors associated with the training effect are suggestive of the potential for body remodeling and improvements in body size, shape, and muscle tone, in addition to a reduction in BMI^38^.

Interventions carried out in camps showed potential effects on the overall self-esteem of adolescents, this is due to the combination of activities developed, with emphasis on nutritional, physical and emotional aspects, a predictor associated with improvements in the level of self-esteem was also weight reduction. In one of the studies that used the camp environment intervention, participants had an average weight reduction of 5.6 kg^50^.

Furthermore, camp-based interventions also achieved significant improvements in the overall self-esteem of obese adolescents, even when there was no significant apparent weight reduction, which may be associated with self-efficacy, such as regular exercise and a supportive environment that can promote the development of skills and greater social interactions, establishing new friendships or being in an environment less vulnerable to experiences associated with stigma and victimization^37^.

Educational interventions have multiple benefits, this occurs due to the nature of these interventions, exploring different skills such as learning, behaviors and attitudes, enabling changes and reflections on self-esteem, moving away from stigmas, teasing from peers and socially defined aesthetic standards, bringing them closer to beliefs and values ​​congruent with self-worth and self-confidence. It is worth noting that the intervention period is crucial to ensure the potential effects. The application of an educational program over a period of 12 months was able to identify improvements in body satisfaction, changes in attitudes and perception related to self-esteem^40^.

The importance of interventions based on familism is also reinforced, in which, in a study carried out with obese Latino adolescents, the family relationship represented a protective factor for self-esteem and, consequently, associated with general well-being. Structured and stable families can provide adolescents with a more comprehensive view of their qualities and values that go beyond weight and physical appearance, providing a support network and skills to develop interpersonal relationships more easily and engage in activities aligned with their goals^25^.

The childhood period plays an important role in the formation of self-esteem in children and adolescents. Sensitive and supportive caregivers contribute to the formation of attachment in children. As they receive positive feedback, they form a positive self-concept, which makes them more secure and with better self-esteem^26^. The self-esteem formed during childhood can continue during adolescence. Thus, the family is one of the most important factors influencing the self-esteem of adolescents, especially among girls^66^. A systematic review identified that multicomponent interventions such as nutrition, education, and physical activity have shown benefits in improving self-esteem, although the relationship between self-esteem and obesity is not fully understood. The adoption of these strategies, focusing on adolescent self-esteem, can be effective in reducing BMI and improving the quality of life of these individuals. Regarding the instruments used to assess self-esteem, two different types of instruments were identified, including: the Rosenberg Self-Esteem Scale and the Harter Self-Perception Profile for Children/Adolescents^11^.

These data corroborate those found in this review, in which the assessment of self-esteem was carried out quantitatively, relying on the use of validated instruments, the most used being the Rosenberg Self-Esteem Scale^11^ ^,^ ^16^ ^-^ ^21^ ^,^ ^25^ ^-^ ^26^ ^,^ ^30^ ^,^ ^32^ ^-^ ^34^ ^,^ ^40^ ^-^ ^41^ ^,^ ^45^ ^,^ ^49^ ^,^ ^53^. The validated and adapted versions for the Portuguese^33^ and Spanish^17^ languages were also used. Next, there is the Coopersmith Self-Esteem Inventory, which assesses self-esteem in different areas: family, academic, social, and general^27^ ^,^ ^41^ ^,^ ^44^ ^,^ ^52^; and the Harter Self-Perception Profile scale for Children/Adolescents, which assesses in more detail aspects of individuals’ lives regarding the global dimension of self-esteem in different dimensions or domains of life^11^ ^,^ ^38^.

The variety of instruments identified in the synthesis of results highlights the complexity of self-esteem assessment, as well as the investigation of this concept in different aspects of the individual, whether in more comprehensive approaches such as global self-esteem or in specific components within self-esteem and how these relate to and impact people’s lives at a psychological and social level, including adolescents.

Cross-sectional studies have been widely used to assess self-esteem among adolescents and correlate it with other variables^17^ ^-^ ^18^ ^,^ ^21^ ^,^ ^23^ ^-^ ^33^ ^,^ ^39^ ^-^ ^40^ ^,^ ^42^ ^,^ ^44^ ^,^ ^52^ ^-^ ^53^. This type of study is useful for determining the prevalence of a condition of interest in a population over a given period of time, identifying associations, generating hypotheses for future investigations, and contributing to the planning of interventions. However, it has weaknesses, such as not allowing the establishment of causal relationships and analysis of the evolution of a given construct over time. Furthermore, it is susceptible to selection biases because the sample of the population of interest may not be representative, compromising the generalization of the results^67^.

In addition, the role of health teams is fundamental for the screening and early identification of adolescents with high levels of stress and low self-esteem. To this end, these professionals can act through school health promotion programs, given that this is an environment accessible to adolescents, and can use reliable and validated instruments, mental health examinations, as well as counseling and school integration projects^23^ ^,^ ^43^.

At this point, it is highlighted that the multidisciplinary nature of the team is essential for building a support network for adolescents with obesity, where each professional plays a key role. The team can be made up of parents, teachers, social workers, counselors and psychologists, establishing a task force capable of promoting a supportive environment that allows adolescents to develop skills, competencies and confidence to explore issues of mental health, obesity and self-esteem^22^ ^-^ ^23^ ^,^ ^43^ ^,^ ^45^.

It is worth noting that nurses are in a favorable position, since, with regard to health promotion at school, it is this professional who establishes prior communication with teachers, parents, students and other staff. By observing and talking to adolescents, it is possible to identify signs of tiredness, depressive mood, discouragement, which can alert nurses to the need to refer this adolescent for a more complete evaluation with other mental health professionals^36^ ^,^ ^41^ ^,^ ^43^.

Another important point is that health professionals need to be aware of the effects of low self-esteem on the mental health of adolescents with obesity, given that early interventions and other therapeutic approaches generally focus on body weight, but should also be aligned with self-esteem, which influences both the adolescent’s well-being and the presence of psychological distress^23^ ^,^ ^45^.

A limitation of this review is the approach used by the included studies. Despite the use of validated instruments to measure self-esteem among adolescents with obesity, it is important to highlight that self-esteem is a complex concept, influenced by different variables and contexts, which should be considered for a more comprehensive assessment. Another limitation refers to the fact that in some studies self-esteem was assessed only at the time of the intervention, and it is necessary to carry out post-intervention assessments, since self-esteem can vary over time and be affected by multiple factors.

The findings of this study contribute to advances in the field of Nursing by deepening the understanding of the self-esteem of adolescents with obesity. Furthermore, identifying the factors associated with low self-esteem and vulnerabilities present in this group, especially the influence of social standards imposed on females, requires preparation and careful observation from this professional. Additionally, the role of the nurse as a health educator is highlighted, guiding potential interventions to promote self-esteem, considering that improvements in this aspect directly impact well-being. This study contributes evidence for clinical practice and more effective and sensitive holistic and multidisciplinary care strategies within the psychosocial context of adolescents.

## Conclusion

Adolescence is a period of change in which psychological fluctuations occur, whether related to mood, emotions, or self-esteem. When associated with health problems, these changes can occur more intensely, resulting in negative impacts on mental health.

The results of some studies included in the review indicate that obesity in adolescents favors low self-esteem, and in some cases, it was considered a predictor for increased BMI. In this regard, it is noteworthy that low self-esteem is frequently identified among the female population, a factor that may be related to social pressures and the influence of peers and the media regarding beauty standards imposed and accepted by society.

Low self-esteem affects different dimensions of the life of an obese adolescent, such as physical, mental, and social. In addition, it increases vulnerabilities to Mental and Eating Disorders, contributing, in more severe cases, to the risk of suicidal behaviors.

It is noteworthy that interventions at the school level and in the daily lives of adolescents, based on the re-education of sedentary habits, physical activity and sports, as well as the emphasis on emotional aspects and the development of skills and abilities, are presented as potential for improving self-esteem. The role of the family is essential, offering support and serving as an example of valuing the uniqueness of the adolescent, perceiving them beyond the diagnosis of obesity, considering their needs, tastes, qualities and expectations.

It is suggested that new studies be carried out, mainly of a qualitative nature, that explore in greater depth and complexity the perception of adolescents with obesity in relation to self-esteem, considering the biopsychosocial dimensions of this population. Investment in research in the area favors the development of intervention strategies that seek to promote the health and well-being of adolescents. 

## Data Availability

All data generated or analysed during this study are included in this published article.
