# Supplementary material for: Self-esteem in adolescents with obesity: a scoping review
Source: Rev Lat Am Enfermagem. 2026 Aug 10;34:e4888. doi: 10.1590/1518-8345.7936.4888 (PMC13459729; doi:10.1590/1518-8345.7936.4888)
Supplement: Translation

# Translation: Autoestima em adolescentes com obesidade: revisão de escopo^*^

**DOI:** 10.1590/1518-8345.7936.4889

**Authors:** Chrisllayne Oliveira da Silva, Nanielle Silva Barbosa, Ana Larissa Gomes Machado, Ítalo Arão Pereira Ribeiro, Ana Maria Ribeiro dos Santos, Márcia Astrês Fernandes

**Affiliations:** 1 Universidade Federal do Piauí, Teresina, PI, Brasil.; 2 Bolsista da Coordenação de Aperfeiçoamento de Pessoal de Nível Superior (CAPES), Brasil.; 3 Universidade Federal de Mato Grosso do Sul, Coxim, MS, Brasil.

**Keywords:** Adolescente, Autoimagem, Obesidade, Bem-Estar Psicológico, Enfermagem, Revisão

## Abstract

**Objetivo:**

mapear as evidências científicas sobre a avaliação, percepção e intervenções direcionadas à autoestima em adolescentes com obesidade.

**Método:**

revisão de escopo, cujo protocolo foi registrado na plataforma *Open Science Framework* com o identificador 10.17605/OSF.IO/C6W72. Foram incluídos estudos primários e secundários, disponíveis na íntegra, sem restrição temporal e de idioma, de oito bases de dados e literatura cinzenta. Cartas ao editor, estudos em fase de protocolo ou ainda sem resultados foram excluídos.

**Resultados:**

foram selecionados 39 estudos para análise. A síntese de evidências sobre a avaliação, percepção e intervenções dos adolescentes com obesidade investigados indicou a baixa autoestima, a qual esteve relacionada ao sexo feminino, à vergonha corporal, à exposição à vitimização por pares, a sintomas depressivos e ao baixo desempenho escolar. As intervenções com potenciais resultados na melhora da autoestima estavam relacionadas à redução do peso, como práticas esportivas, programas educativos e apoio familiar.

**Conclusão:**

existe uma relação complexa entre a obesidade em adolescentes e a baixa autoestima, tornando fundamental o estabelecimento de boas práticas alimentares e atividades físicas regulares, além do suporte emocional aos adolescentes considerando as dimensões biopsicossociais.

## Destaques

**(1)** Adolescentes com obesidade apresentam menor autoestima. **(2)** A baixa autoestima afeta o bem-estar físico e mental e as relações sociais. **(3)** O apoio familiar é um dos fatores que interferem na formação da autoestima. **(4)** Boas práticas alimentares e atividades físicas regulares favorecem a autoestima.

## Introdução

A adolescência é geralmente considerada uma fase de transição, embora o indivíduo não seja visto como criança, ainda não é considerado um adulto. É justamente nesse período que o desenvolvimento humano ocorre de forma acelerada, seja em aspectos físicos, cognitivos, sociais, sexuais e emocionais^1^. Essas mudanças intensas podem contribuir para situações de riscos à saúde desse(a) adolescente e para o desenvolvimento de Doenças Crônicas Não Transmissíveis (DCNT) como a obesidade, que é o acúmulo excessivo ou anormal de gordura corporal^1^ ^-^ ^3^.

Os adolescentes com obesidade lidam constantemente com fatores externos, como dificuldade de aceitação e inclusão em grupos, estigma social, *bullying*, que podem resultar em baixos níveis de autoestima, levando a um quadro de vulnerabilidade para transtornos alimentares, ansiedade, depressão e isolamento social^4^ ^-^ ^5^.

Diante do exposto, a autoestima dos adolescentes deve ser considerada como uma variável relevante para avaliar a saúde desses indivíduos, tendo em vista que está relacionada à construção da identidade e da adaptação ao meio, funcionando como um dos reguladores de bem-estar^6^ ^-^ ^7^.

Em uma pesquisa de base nacional com estudantes brasileiros de 12 a 17 anos, três em cada dez estudantes apresentam insatisfação com a imagem corporal, enquanto os adolescentes com obesidade são propensos à baixa autoestima e negligência em relação à autopercepção associada ao excesso de peso e riscos de comorbidades^2^.

Nessa vertente, a autoestima, definida como uma autoavaliação sobre si mesmo, sobre as próprias atitudes e a relação com os demais, interfere em como o indivíduo reage às mudanças internas e externas, além de atribuir valores e sentimentos favoráveis ou desfavoráveis que, quando negativos, repercutem na saúde do adolescente. Por outro lado, em níveis satisfatórios, podem influenciar na adoção de atitudes de promoção da saúde e proteção a comportamentos prejudiciais^6^ ^,^ ^8^ ^-^ ^9^.

A partir de uma busca prévia na literatura, foram identificados dois estudos sobre a relação entre obesidade e autoestima: uma revisão de escopo que mapeou os instrumentos existentes para mensurar a autoestima em crianças e adolescentes e uma revisão sistemática que avaliou se intervenções com multicomponentes teriam impacto na autoestima dos adolescentes^10^ ^-^ ^11^. Assim, o estudo se diferencia dos demais por abordar os aspectos referentes à percepção e conceitos dos adolescentes com obesidade diante da autoestima e como isso repercute na vida cotidiana, além de compreender a relação entre obesidade e autoestima como um fator de adoecimento ou protetivo.

Apesar do significativo número de estudos sobre a obesidade na adolescência em diferentes bases de dados, ainda existe uma lacuna em relação à autoestima, que é um fator importante no desenvolvimento pessoal e manutenção das condições de saúde.

Dessa forma, compreender a percepção dos adolescentes com obesidade pode fornecer evidências relevantes relacionadas à autoimagem, tendo em vista que a obesidade pode constituir um fator de risco para um autoconceito negativo, podendo ser um agravante para uma baixa autoestima. Além disso, as informações obtidas podem contribuir para o desenvolvimento de futuras pesquisas que possam direcionar intervenções com ênfase nas complexidades e na mitigação do estigma associado à obesidade e sua repercussão na saúde mental.

Este estudo teve como objetivo mapear as evidências científicas sobre a avaliação, percepção e intervenções direcionadas à autoestima em adolescentes com obesidade.

## Método

### Desenho do estudo

Estudo de revisão da literatura, do tipo revisão de escopo, conduzido de acordo com as etapas recomendadas pelo JBI: título e perguntas de revisão, critérios de inclusão, estratégia de busca, triagem e seleção de evidências, extração e análise de dados e apresentação dos resultados; e relatada utilizando as diretrizes do *Preferred Reporting Items for Systematic Reviews and Meta-Analyses extension for Scoping Review* (PRISMA-ScR)^12^ ^-^ ^13^ *.*

O protocolo de revisão foi registrado no *Open Science Framework* (OSF) com identificador DOI 10.17605/OSF.IO/ C6W72. Anteriormente à elaboração do protocolo, os autores verificaram na literatura a existência de outras revisões com tema e objetivos semelhantes aos desta revisão. Foram encontradas duas revisões^10^ ^-^ ^11^, porém foram direcionadas aos instrumentos de avaliação da autoestima e a intervenções específicas, diferenciando-se dos objetivos do presente estudo.

### Cenário

As bases de dados consultadas foram: Literatura Latino-americana e do Caribe em Ciências da Saúde (LILACS), Base de Dados de Enfermagem (BDENF), Índice Bibliográfico Espanhol em Ciências da Saúde (IBECS), Index Psicologia (INDEXPSI), via Biblioteca Virtual em Saúde (BVS), *Medical Literature Analysis and Retrieval System Online* (MEDLINE) via PubMed, *Web of Science*, *Scopus* e APA PsycInfo^®^.

A literatura cinzenta foi recuperada em fontes como Biblioteca Digital Brasileira de Teses e Dissertações (BDTD), Catálogo de Teses e Dissertações (CTD) da Coordenação de Aperfeiçoamento de Pessoal de Nível Superior (CAPES) e Google *Scholar* (nas 10 primeiras páginas).

### Período

A pesquisa foi realizada entre dezembro de 2024 e janeiro de 2025.

### População

O acrônimo PCC foi utilizado, sendo a População os adolescentes, caracterizados como indivíduos com idade entre 10 e 19 anos, conforme definição da Organização Mundial da Saúde (OMS). O Conceito adotado foi autoestima e o Contexto de interesse foi obesidade. A pergunta de revisão foi: quais as evidências disponíveis sobre a avaliação, percepção e intervenções direcionadas à autoestima em adolescentes com obesidade? 

### Critérios de seleção

Foram incluídos os estudos primários e secundários sobre autoestima em adolescentes com obesidade, tanto estudos que consideraram essas duas variáveis como principais quanto aqueles que analisaram diferentes variáveis, mas que verificaram autoestima em adolescentes com a comorbidade, primários e secundários, disponíveis na íntegra, sem restrição temporal e de idioma. Cartas ao editor, estudos em fase de protocolo ou ainda sem resultados foram excluídos. 

### Definição da amostra

Uma busca inicial sobre a temática de interesse foi realizada na literatura, com auxílio de uma bibliotecária. A partir dos estudos recuperados, realizou-se uma análise dos termos contidos no título, resumo, texto e descritores/palavras-chaves a fim de selecioná-los nos Descritores em Ciências da Saúde (DeCS) e *Medical Subject Headings* (MeSH). Esses termos foram combinados entre si, de forma a garantir o maior retorno da quantidade de estudos, estruturando as estratégias de busca específicas a cada base de dados, conforme a Figura 1. As estratégias foram testadas por cada uma das duas revisoras e pela bibliotecária, de forma independente, a fim de garantir o mesmo resultado em todas as buscas.

**Figura 1:** Estratégia de busca em bases de dados e literatura cinzenta. Teresina, PI, Brasil, 2024

Base de dados	Estratégia
LILACS*/BDENF^†^/IBECS^‡^/INDEXPSI^§^ (BVS^ǁ^)	((mh:(adolescente )) OR (adolescentes) AND (adolescência)) AND ((mh:(autoimagem)) OR (autoestima)) AND ((mh:(obesidade)) OR (obesidade)) AND db:(“LILACS*” OR “IBECS^‡^” OR “BDENF^†^” OR “INDEXPSI^§^”) AND instance:”regional”
MEDLINE^¶^ (PubMed)	(((“self concept”[MeSH Terms]) OR (“self esteem”[All Fields])) AND (((“adolescent”[MeSH Terms]) OR (“adolescents”[All Fields])) OR (“adolescence”[All Fields]))) AND ((Obesity[MeSH Terms]) OR (“obesity”[All Fields]))
Web of Science	((ALL=(Adolescent) OR ALL=(Adolescents) OR ALL=(Adolescence)) AND ALL=(Obesity)) AND (ALL=(‘’Self Concept’’) OR ALL=(‘’Self Esteem’’))
Scopus	( ( TITLE-ABS-KEY ( adolescent ) OR TITLE-ABS-KEY ( adolescents ) OR TITLE-ABS-KEY ( adolescence ) ) ) AND ( TITLE-ABS-KEY ( obesity ) ) AND ( ( TITLE-ABS-KEY ( ‘’self concept’’ ) OR TITLE-ABS-KEY ( self esteem ) ) )
APA** PsycInfo^®^	((Any Field: (Adolescent)) OR (Any Field: (Adolescents)) OR (Any Field: (Adolescence))) AND ((Any Field: (Self concept)) OR (Any Field: (Self Esteem))) AND ((Any Field: (Obesity)))
**Literatura cinzenta**	**Estratégia**
BDTD^††^	(Adolescentes OR adolescente OR adolescência) AND (Autoimagem OR Autoestima) AND (Obesidade)
CTD-CAPES^‡‡^	(Adolescentes OR adolescente OR adolescência) AND (Autoimagem OR Autoestima) AND (Obesidade)
Google *Scholar*	Adolescents AND ‘’Self esteem’’ AND Obesity

*LILACS = Literatura Latino-americana e do Caribe em Ciências da Saúde; ^†^BDENF = Base de Dados de Enfermagem; ^‡^IBECS = Índice Bibliográfico Espanhol em Ciências da Saúde; ^§^INDEXPSI = Index Psicologia; ^ǁ^BVS = Biblioteca Virtual em Saúde; ^¶^MEDLINE = *Medical Literature Analysis and Retrieval System Online;* **APA = *American Psychological Association*; ^††^BDTD = Biblioteca Digital Brasileira de Teses e Dissertações; ^‡‡^CTD-CAPES = Catálogo de Teses e Dissertações da Coordenação de Aperfeiçoamento de Pessoal de Nível Superior

### Coleta de dados

A busca, seleção e análise foi realizada de forma independente, às cegas, por duas revisoras, autoras do estudo, uma com titulação de Mestra em Ciências e Saúde e com conhecimento na área de Saúde da Criança e Adolescente e a outra Mestra em Enfermagem e com conhecimento na área de Saúde Mental, ambas com experiência na elaboração e publicação de revisões. As divergências quanto à inclusão ou não de determinado texto, após discussão entre as duas primeiras revisoras, foram resolvidas por uma terceira revisora, enfermeira Doutora em Enfermagem com conhecimento na área de Saúde Coletiva.

As citações identificadas foram importadas para o gerenciador de referências Rayyan^®^, desenvolvido pelo *Qatar Computing Research Institute* (QCRI) para retirada de duplicidades, seleção e triagem.

A primeira fase de seleção compreendeu a leitura de títulos e resumos. Estudos que atenderam aos critérios de inclusão foram analisados em uma segunda fase, via leitura dos manuscritos na íntegra. Os motivos para a exclusão dos estudos foram registrados e relatados em fluxograma^14^.

### Variáveis do estudo

As variáveis extraídas dos estudos foram: autoria, país de origem, idioma, ano de publicação, objetivo, tipo de estudo, amostra, local da coleta de dados, instrumento de avaliação da autoestima, principais resultados e nível de evidência^15^.

### Instrumento de coleta de dados

As informações foram mapeadas conforme recomendações do JBI para caracterização das fontes de evidência, registradas e organizadas em formato de quadro sinóptico elaborado no Microsoft Office Excel 2024.

### Processamento e análise de dados 

A análise dos dados foi realizada de forma descritiva, quantitativa e qualitativa. A síntese dos resultados foi apresentada de forma narrativa e por meio de figuras. 

### Aspectos éticos

O estudo utilizou dados secundários, portanto não houve apreciação pelo Comitê de Ética em Pesquisa (CEP).

## Resultados

Foram identificados 6860 estudos nas bases de dados, dos quais 2474 foram excluídos por duplicata e 4386 seguiram para leitura do título e resumo. Após a primeira leitura, 50 foram selecionados para leitura na íntegra, dos quais 36 foram incluídos na amostra final. A partir da literatura cinzenta, três estudos foram incluídos. Assim, 39 estudos compuseram a amostra final da revisão (Figura 2).

A Figura 3 mostra a descrição dos estudos segundo o país de origem, ano de publicação, idioma, tipo de estudo, nível de evidência, amostra, local e instrumento de avaliação da autoestima.

**Figura 2:** Fluxograma de seleção dos estudos. Teresina, PI, Brasil, 2024

*BVS = Biblioteca Virtual em Saúde; ^†^MEDLINE = *Medical Literature Analysis and Retrieval System Online*; ^‡^APA = *American Psychological Association*; ^§^BDTD = Biblioteca Digital Brasileira de Teses e Dissertações; ^ǁ^CTD-CAPES = Catálogo de Teses e Dissertações da Coordenação de Aperfeiçoamento de Pessoal de Nível Superior

**Figura 3:** Descrição dos estudos selecionados quanto ao país de origem, ano de publicação, idioma, tipo de estudo, nível de evidência, amostra, local e instrumento de avaliação da autoestima. Teresina, PI, Brasil, 2024

Ordem	País de origem/ano de publicação/idioma	Método
01^16^	Brasil/2024/Inglês	Tipo de estudo/Nível de evidência: ensaio clínico randomizado controlado/II Amostra: 40 adolescentes receberam a intervenção e 13 participaram do grupo controle, com idade entre 10 e 17 anos. Local: centro de estudos. Instrumento: Escala de Autoestima de Rosenberg.
02^17^	Chile/2024/Espanhol	Tipo de estudo/Nível de evidência: transversal/VI Amostra: 397 adolescentes na faixa etária de 10 a 19 anos. Local: escolas. Instrumento: Escala de Autoestima de Rosenberg.
03^18^	Marrocos/2024/Inglês	Tipo de estudo/Nível de evidência: transversal/VI Amostra: 410 adolescentes com idade entre 12 e 17 anos. Local: escolas. Instrumento: Escala de Autoestima de Rosenberg.
04^19^	Turquia/2023/Inglês	Tipo de estudo/Nível de evidência: caso-controle/IV Amostra: 50 crianças com diagnóstico de obesidade e 48 participantes no grupo controle, com idade entre 11 e 17 anos. Local: universidade. Instrumento: Escala de Autoestima de Rosenberg.
05^20^	Reino Unido/2023/Inglês	Tipo de estudo/Nível de evidência: coorte prospectivo/IV Amostra: 18.818 participantes de 11 a 17 anos. Local: não especificado. Instrumento: Escala de Autoestima de Rosenberg.
06^21^	Reino Unido/2022/Inglês	Tipo de estudo/Nível de evidência: transversal/VI Amostra: 9046 adolescentes de 11 e 14 anos Local: residências. Instrumento: Escala de Autoestima de Rosenberg.
07^22^	China/2021/Inglês	Tipo de estudo/Nível de evidência: caso-controle/IV Amostra: 128 estudantes com obesidade e 128 com peso normal. Local: escola. Instrumento: Escala de Estereótipo Corporal para Adolescentes e Adultos.
08^23^	Estados Unidos da América/2021/Inglês	Tipo de estudo/Nível de evidência: transversal/VI Amostra: 6.748 adolescentes. Local: escolas. Instrumento: Versão modificada do Inventário de Autoestima de Rosenberg.
09^24^	Grécia/2020/Inglês	Tipo de estudo/Nível de evidência: transversal/VI Amostra: 528 adolescentes com idade entre 10 e 12 anos. Local: escola. Instrumento: Perfil de Autopercepção para Crianças.
10^25^	Estados Unidos da América/2020/Inglês	Tipo de estudo/Nível de evidência: transversal/VI Amostra: 160 adolescentes de 14 a 16 anos. Local: centros comunitários. Instrumento: Escala de Autoestima de Rosenberg.
11^26^	Irã/2020/Inglês	Tipo de estudo/Nível de evidência: transversal/VI Amostra: 173 adolescentes com idade entre 12 e 16 anos. Local: centro de estudos. Instrumento: Escala de Autoestima de Rosenberg.
12^27^	Chile/2019/Espanhol	Tipo de estudo/Nível de evidência: transversal/VI Amostra: 419 adolescentes entre 14 e 17 anos. Local: escola. Instrumento: Inventário de Autoestima Coopersmith.
13^28^	Egito/2019/Inglês	Tipo de estudo/Nível de evidência: transversal/VI Amostra: 533 adolescentes de 10 a 19 anos. Local: escola. Instrumento: Escala de Autoestima de Rosenberg.
14^29^	Chile/2018/Inglês	Tipo de estudo/Nível de evidência: transversal/VI Amostra: 712 escolares de 10 a 13 anos. Local: escola. Instrumento: TAE-student*.
15^30^	Espanha/2018/Espanhol	Tipo de estudo/Nível de evidência: transversal/VI Amostra: 364 escolares entre 11 e 13 anos de idade. Local: centro educativos públicos. Instrumento: TAE-student*.
16^31^	Chile/2018/Inglês	Tipo de estudo/Nível de evidência: transversal/VI Amostra: 656 adolescentes entre 11 e 14 anos. Local: escola. Instrumento: TAE-student*.
17^32^	Índia/2017/Inglês	Tipo de estudo/Nível de evidência: transversal/VI Amostra: 150 adolescentes com média de idade de 14,5 anos. Local: escola. Instrumento: Escala de Autoestima de Rosenberg.
18^33^	Brasil/2017/Português	Tipo de estudo/Nível de evidência: transversal/VI Amostra: 418 adolescentes de 14 e 18 anos. Local: escola. Instrumento: Escala de Autoestima de Rosenberg.
19^34^	Estados Unidos da América/2017/Inglês	Tipo de estudo/Nível de evidência: coorte prospectivo/IV Amostra: 139 adolescentes com idade entre 13 e 18 anos. Local: hospital. Instrumento: Perfil de Autopercepção para Adolescentes.
20^11^	Austrália/2017/Inglês	Tipo de estudo/Nível de evidência: revisão sistemática e metanálises/I Amostra: treze estudos com 1.157 adolescentes com sobrepeso ou obesos, com idades entre 10 e 19 anos. Local: escola e residências. Instrumento: Escala de Autoestima de Rosenberg; Perfil de Autopercepção de Harter para Crianças; Autoestima Global de Marsh.
21^35^	Arábia Saudita/2016/Árabe	Tipo de estudo/Nível de evidência: coorte retrospectivo/IV Amostra: 257 adolescentes com idade média de 18 anos. Local: escola. Instrumento: Escala de Autoestima de Rosenberg.
22^36^	Itália/2016/Inglês	Tipo de estudo/Nível de evidência: caso-controle/IV Amostra: 111 estudantes com obesidade e 111 com peso normal, na faixa etária de 13 a 19 anos. Local: escola. Instrumento: Escala de Autoestima de Rosenberg.
23^37^	Reino Unido/2016/Inglês	Tipo de estudo/Nível de evidência: ensaio clínico randomizado controlado/II Amostra: 303 jovens com idade entre 11 e 17 anos. Local: residências. Instrumento: Perfil de Autopercepção para Crianças.
24^38^	Canadá/2015/Inglês	Tipo de estudo/Nível de evidência: ensaio clínico randomizado controlado/II Amostra: 304 participantes de 14 a 18 anos. Local: instalações comunitárias. Instrumento: Perfil de Autopercepção Física para Crianças; Subescala Harter de Autoestima Global.
25^39^	Espanha/2015/Espanhol	Tipo de estudo/Nível de evidência: transversal/IV Amostra: 89 adolescentes com idade entre 12 e 16 anos. Local: hospital. Instrumento: Perfil de Saúde e Doença Infantil.
26^40^	Espanha/2015/Espanhol	Tipo de estudo/Nível de evidência: transversal/VI Amostra: 296 crianças com idade entre 12 e 14 anos. Local: escola. Instrumento: Escala de Autoestima de Rosenberg.
27^41^	Estados Unidos da América/2014/Inglês	Tipo de estudo/Nível de evidência: caso-controle/IV Amostra: 1419 estudantes adolescentes. Local: escola. Instrumento: Escala Geral de Autoestima.
28^42^	Irã/2014/Inglês	Tipo de estudo/Nível de evidência: transversal/VI Amostra: 8.510 adolescentes com idades entre 15 e 17 anos. Local: escolas. Instrumento: Questionário de Relações Multidimensionais Corpo-Eu; Inventário de Autoestima de Coopersmith.
29^43^	Estados Unidos da América/2012/Inglês	Tipo de estudo/Nível de evidência: caso-controle/IV Amostra: 101 adolescentes com idade entre 14 e 18. Local: escola. Instrumento: Escala de Autoestima de Rosenberg.
30^44^	Estados Unidos da América/2012/Inglês	Tipo de estudo/Nível de evidência: transversal/IV Amostra: 119 adolescentes com idade entre 11 e 18 anos. Local: hospital. Instrumento: Inventário de Autoestima de Coopersmith.
31^45^	Estados Unidos da América/2011/Inglês	Tipo de estudo/Nível de evidência: coorte prospectivo/IV Amostra: 806 adolescentes. Local: escola. Instrumento: Escala de Autoestima de Rosenberg.
32^46^	Estados Unidos da América/2010/Inglês	Tipo de estudo/Nível de evidência/IV Amostra: 4458 adolescentes com idade entre 12 e 16 anos Local: remoto. Instrumento: Perfil de Autoestima de Harter para Crianças.
33^47^	Suécia/2010/Inglês	Tipo de estudo/Nível de evidência: coorte prospectivo/IV Amostra: uma coorte dos anos 2000 com 191 adolescentes e uma coorte de 2008 com 170 adolescentes. Local: escola. Instrumento: Perfil de Autopercepção Física para Crianças e Adolescentes.
34^48^	França/2010/Francês	Tipo de estudo/Nível de evidência: ensaio clínico randomizado controlado/II Amostra: 16 adolescentes com média de idade de 14,9 anos. Local: hospital. Instrumento: Inventário Físico de Si Mesmo.
35^49^	Turquia/2004/Inglês	Tipo de estudo/Nível de evidência: caso-controle/IV Amostra: 90 adolescentes com idade entre 12 e 16 anos. Local: escola. Instrumento: Escala de Autoestima de Rosenberg.
36^50^	Reino Unido/2023/Inglês	Tipo de estudo/Nível de evidência: ensaio clínico randomizado controlado/II Amostra: 57 adolescentes obesos e 38 adolescentes com peso normal. Local: acampamento residencial. Instrumento: Perfil de Autopercepção para Crianças.
37^51^	Suécia/1999/Inglês	Tipo de estudo/Nível de evidência: caso-controle/IV Amostra: 58 adolescentes com obesidade e 58 com peso normal, com idades entre 14 e 18 anos. Local: escola. Instrumento: Questionário Eu Penso, Eu Sou.
38^52^	França/1995/Inglês	Tipo de estudo/Nível de evidência: transversal/VI Amostra: 93 adolescentes com idade entre 13 e 19 anos. Local: hospital. Instrumento: Inventário de Autoestima de Coopersmith.
39^53^	Estados Unidos da América/1988/Inglês	Tipo de estudo/Nível de evidência: transversal/VI Amostra: 550 meninas de 14 e 16 anos. Local: escola. Instrumento: Escala de Autoestima de Rosenberg.

*TAE-student = Teste de Autoestima Escolar

Quanto ao país de origem dos estudos, nove foram realizados nos Estados Unidos da América^23^ ^,^ ^25^ ^,^ ^34^ ^,^ ^41^ ^,^ ^43^ ^-^ ^46^ ^,^ ^53^, quatro no Chile^17^ ^,^ ^27^ ^-^ ^28^ ^,^ ^31^ e Reino Unido^20^ ^-^ ^21^ ^,^ ^37^ ^,^ ^50^, três na Espanha^29^ ^,^ ^39^ ^-^ ^40^, dois no Brasil^16^ ^,^ ^33^, França^48^ ^,^ ^52^, Irã^26^ ^,^ ^42^, Suécia^47^ ^,^ ^51^ e Turquia^19^ ^,^ ^49^ e um na Arábia Saudita^35^, Austrália^11^, Canadá^38^, China^22^, Egito^30^, Índia^32^, Itália^36^, Grécia^24^ e Marrocos^18^. Um mapa representativo dos países de origem das publicações (Figura 4).

**Figura 4:** País de origem e número de publicações correspondentes. Teresina, PI, Brasil, 2024

Referente ao ano de publicação, quatro foram publicados em 2017^11^ ^,^ ^32^ ^-^ ^34^, três em 2024^16^ ^-^ ^18^, 2020^24^ ^-^ ^26^, 2018^29^ ^-^ ^31^, 2016^35^ ^-^ ^37^, 2015^38^ ^-^ ^40^, e 2010^46^ ^-^ ^48^, dois em 2023^19^ ^-^ ^20^, 2021^22^ ^-^ ^23^, 2019^27^ ^-^ ^28^, 2014^31^ ^,^ ^42^ ^)^ e 2012^43^ ^-^ ^44^, um em 2022^21^, 2011^45^, 2004^49^, 2003^50^, 1999^51^, 1995^52^ e 1988^53^. Quanto ao idioma, a maioria dos estudos foi publicada em inglês^11^ ^,^ ^16^ ^,^ ^18^ ^-^ ^26^ ^,^ ^28^ ^-^ ^29^ ^,^ ^31^ ^-^ ^32^ ^,^ ^34^ ^,^ ^36^ ^-^ ^38^ ^,^ ^41^ ^-^ ^47^ ^,^ ^49^ ^-^ ^53^, seguindo por espanhol^17^ ^,^ ^27^ ^,^ ^20^ ^,^ ^39^ ^-^ ^40^, português^33^, árabe^35^ e francês^48^.

Os tipos de estudos desenvolvidos foram transversais^17^ ^-^ ^18^ ^,^ ^21^ ^,^ ^23^ ^-^ ^33^ ^,^ ^39^ ^-^ ^40^ ^,^ ^42^ ^,^ ^44^ ^,^ ^52^ ^-^ ^53^, caso-controle^19^ ^,^ ^22^ ^,^ ^36^ ^,^ ^41^ ^,^ ^43^ ^,^ ^50^ ^,^ ^52^, coorte^20^ ^,^ ^34^ ^,^ ^36^ ^,^ ^46^ ^-^ ^48^, ensaios clínicos randomizados controlados^16^ ^,^ ^38^ ^-^ ^39^ ^,^ ^49^ ^,^ ^51^ e uma revisão sistemática^11^, com amostra variando entre 16^48^ e 18818^20^ adolescentes. O nível de evidência foi classificado, de forma que se identificaram estudos de nível VI^17^ ^-^ ^18^ ^,^ ^21^ ^,^ ^23^ ^-^ ^33^ ^,^ ^39^ ^-^ ^40^ ^,^ ^42^ ^,^ ^44^ ^,^ ^52^ ^-^ ^53^, IV^19^ ^-^ ^20^ ^,^ ^22^ ^,^ ^34^ ^,^ ^36^ ^,^ ^41^ ^,^ ^43^ ^,^ ^46^ ^-^ ^48^ ^,^ ^50^ ^,^ ^52^, II^16^ ^,^ ^38^ ^-^ ^39^ ^,^ ^49^ ^,^ ^51^ ^)^ e I^11^. Os estudos foram realizados em diferentes cenários como acampamento^50^, centros comunitários^25^, centro de estudos^26^, centros educacionais públicos^29^, escolas^11^ ^,^ ^17^ ^-^ ^18^ ^,^ ^22^ ^-^ ^24^ ^,^ ^27^ ^-^ ^28^ ^,^ ^30^ ^-^ ^33^ ^,^ ^35^ ^-^ ^36^ ^,^ ^40^ ^-^ ^43^ ^,^ ^45^ ^,^ ^47^ ^,^ ^49^ ^,^ ^51^ ^,^ ^53^, hospitais^33^ ^,^ ^39^ ^,^ ^44^ ^,^ ^48^ ^,^ ^52^, instalações comunitárias^38^, remoto^46^, residências^11^ ^,^ ^21^ ^,^ ^37^ e universidade^19^, sendo que em um deles não foi especificado^20^.

Os instrumentos Autoestima Global de Marsh^11^, Escala de Autoestima de Rosenberg^11^ ^,^ ^16^ ^-^ ^21^ ^,^ ^25^ ^-^ ^26^ ^,^ ^28^ ^,^ ^30^ ^,^ ^32^ ^-^ ^33^ ^,^ ^36^ ^,^ ^40^ ^,^ ^41^ ^,^ ^45^ ^,^ ^49^ ^,^ ^53^ e versão modificada^23^, Escala de Estereótipo Corporal para Adolescentes e Adultos^22^, Escala Geral de Autoestima^41^, Inventário de Autoestima Coopersmith^27^ ^,^ ^42^ ^,^ ^44^ ^,^ ^52^, Inventário Físico de Si Mesmo^48^, Perfil de Autoestima de Harter para Crianças^46^, Perfil de Autopercepção de Harter para Crianças^11^, Perfil de Autopercepção Física para Crianças^38^, Perfil de Autopercepção Física para Crianças e Adolescentes^47^, Perfil de Autopercepção para Adolescentes^34^, Perfil de Autopercepção para Crianças^24^ ^,^ ^37^ ^,^ ^50^, Perfil de Saúde e Doença Infantil^39^, Questionário Eu Penso, Eu Sou^51^, Questionário de Relações Multidimensionais Corpo-Eu^42^, Subescala Harter de Autoestima Global^38^ e *TAE-student*: teste de autoestima escolar^29^ ^-^ ^31^ foram utilizados para avaliar a autoestima entre os participantes. 

Os estudos identificaram que a obesidade e o aumento do Índice de Massa Corporal (IMC) influenciam na baixa autoestima entre adolescentes, destacando essa prevalência entre o sexo feminino^11^ ^,^ ^17^ ^-^ ^24^ ^,^ ^27^ ^-^ ^30^ ^,^ ^33^ ^,^ ^39^ ^-^ ^43^ ^,^ ^45^ ^-^ ^47^ ^,^ ^51^ ^-^ ^53^. Aponta-se que a baixa autoestima contribui para a vergonha corporal^22^ ^,^ ^36^, um dos preditores para Transtornos Alimentares, bem como sofre influência da exposição à vitimização por pares^41^ ^,^ ^44^, reflete em impactos negativos na saúde mental e no bem-estar^19^ ^-^ ^21^ ^,^ ^26^ ^,^ ^32^ ^,^ ^41^ ^,^ ^43^ ^,^ ^48^ ^-^ ^49^ e desempenho escolar^27^ ^,^ ^34^ ^,^ ^35^. Destaca-se a necessidade de intervenções para a promoção da autoestima nesse público, como a prática esportiva e de atividade física e a importância do apoio familiar^16^ ^,^ ^25^ ^-^ ^26^ ^,^ ^37^ ^-^ ^38^ ^,^ ^40^ ^,^ ^50^. Os objetivos e principais resultados dos estudos são descritos na Figura 5.

**Figura 5:** Descrição dos estudos selecionados quanto aos objetivos e principais resultados. Teresina, PI, Brasil, 2024

Ordem	Objetivos	Principais resultados
01^16^	Analisar os efeitos de 16 semanas de prática de Muay Thai na percepção corporal, autoestima e QV* em adolescentes com sobrepeso/obesidade.	Foi observado aumento na prevalência de autoestima entre os participantes do grupo que realizou práticas de Muay Thai.
02^17^	Relacionar a insatisfação corporal com autoestima, depressão e IMC^†^ em adolescentes.	Da amostra, 17,1% eram obesos e 1,5% eram gravemente obesos. Observou-se a prevalência de baixa autoestima entre o sexo feminino. Foram encontradas correlações entre as variáveis de insatisfação corporal e o escore z do IMC^†^, sintomas depressivos e autoestima.
03^18^	Avaliar os níveis de autoestima, depressão e sua associação com obesidade/excesso de peso.	Na categoria de obesidade, as meninas tinham uma autoestima significativamente mais baixa em relação aos meninos. Foi encontrada uma forte associação entre depressão e obesidade/excesso de peso e baixa autoestima.
04^19^	Investigar os efeitos da obesidade na ansiedade, depressão, autoestima e regulação emocional em crianças e adolescentes	As análises secundárias mostraram que crianças com obesidade tinham autoestima mais baixa do que as com sintomas de ansiedade e depressão. Também foi encontrada correlação entre sintomas de depressão, ansiedade e dificuldades na regulação emocional no grupo com obesidade.
05^20^	Determinar os caminhos de intervenção entre a saúde mental e os sintomas do escore z do IMC^†^ ao longo da adolescência.	Foram encontradas evidências de que a relação entre IMC^†^ e saúde mental foi parcialmente atribuível à felicidade com a aparência e autoestima. Para meninos e meninas, sintomas emocionais elevados em comparação com sintomas baixos/moderados e aumento no escore z do IMC^†^ aos 11 anos foi associado a maiores chances de infelicidade com a aparência e baixa autoestima aos 14 anos.
06^21^	Investigar características específicas de gênero associadas à baixa participação esportiva entre adolescentes do Reino Unido e avaliar o rastreamento do IMC^†^ específico de gênero e associações específicas de gênero entre IMC^†^ e autoestima com base em diferentes níveis de participação esportiva de adolescentes.	Meninos e meninas no quartil mais baixo de participação esportiva eram mais propensos a ter sobrepeso/obesidade e baixa autoestima em comparação com adolescentes no quartil mais alto de participação esportiva.
07^22^	Explorar como a Inteligência Emocional, o gênero e o tamanho corporal influenciam a relação entre a autoestima corporal e o risco de Transtornos Alimentares entre adolescentes.	O gênero e o tamanho corporal influenciaram diretamente a autoestima corporal e o risco de Transtornos Alimentares. Ademais, a Inteligência Emocional atuou como moderadora entre a autoestima corporal e o risco de transtornos.
08^23^	Ampliar a literatura ao utilizar uma medida relativa do peso corporal ou a classificação do IMC^†^ de um aluno dentro de seu grupo de pares, relevante para examinar o impacto do peso corporal nos resultados dos jovens.	Em estudantes do sexo feminino com alto IMC^†^ há probabilidade de experimentar a diminuição na autoestima e um aumento nos sintomas depressivos.
09^24^	Investigar o excesso de peso corporal e a obesidade abdominal em relação a autopercepção, autoestima e ansiedade.	Participantes com sobrepeso e obesidade apresentaram escores mais baixos em todos os domínios da autoestima global em comparação com crianças com peso normal.
10^25^	Examinar o papel de fatores culturais (por exemplo, familismo) e psicossociais (por exemplo, autoestima) como preditores da QV* específica do peso entre adolescentes latinos com obesidade.	O familismo e a autoestima positiva podem servir como fatores de proteção associados a uma maior QV* de adolescentes com obesidade, enquanto a autodepreciação pode ser um fator de risco para uma menor QV* associada ao peso.
11^26^	Investigar o impacto da comunicação e função familiar, e satisfação com a imagem corporal, no bem-estar psicológico considerando: o papel mediador da autoestima e da depressão.	Os resultados mostraram que a comunicação e a função familiar afetaram diretamente o bem-estar psicológico dos adolescentes. Além disso, a comunicação e a função familiar, assim como a satisfação com a imagem corporal, afetaram indiretamente o bem-estar psicológico por meio da autoestima e da depressão.
12^27^	Determinar a associação entre os níveis nutricionais, aptidão física, autoestima e QV* com desempenho acadêmico em adolescentes.	Escolares com obesidade apresentaram menor QVRS^‡^ do que seus pares com sobrepeso e peso normal. Os escolares com sobrepeso e obesidade apresentaram menores níveis de autoestima e esteve relacionado a um baixo desempenho acadêmico.
13^28^	Medir a prevalência de sobrepeso e obesidade e identificar sua relação com a autoestima entre adolescentes escolares.	Resultados do estudo mostram que 71 (13,3%) adolescentes são obesos. Além disso, foi identificado associação estatisticamente significativa entre autoestima e IMC^†^ em que a baixa autoestima estava presente em cerca de três quartos dos adolescentes com sobrepeso e obesidade.
14^29^	Determinar se há associação entre insatisfação com a imagem corporal com parâmetros antropométricos, status de peso e autoestima em crianças de escolas públicas.	A insatisfação com a imagem corporal foi maior entre meninas e crianças com obesidade, que também apresentaram menor autoestima.
15^30^	Relacionar a condição de sobrepeso e obesidade com os níveis de autoestima e felicidade em escolares de centros educacionais públicos.	A autoestima escolar foi significativamente maior nos meninos. O excesso de peso foi associado à infelicidade em crianças em idade escolar.
16^31^	Avaliar a percepção de escolares obesos quanto à sua participação nas aulas de educação física e determinar sua autoestima em comparação com os grupos com sobrepeso e peso normal.	Os escolares com obesidade sentem-se excluídos das aulas de educação física e apresentam baixos níveis de autoestima, comparado com escolares com peso normal.
17^32^	Examinar se as adolescentes com obesidade/sobrepeso diferem das adolescentes com peso normal em termos de autoestima.	A análise correlacional dos dados revelou uma relação negativa do IMC^†^ com a autoestima. As descobertas sugeriram que há necessidade de melhorar a autoestima das adolescentes, que geralmente diminui devido à percepção do peso corporal, bem como devido aos fatores sociais e psicológicos envolvidos.
18^33^	Investigar a autoestima, a imagem corporal e a depressão de adolescentes em diferentes estados nutricionais.	Meninos apresentaram maior prevalência de sobrepeso e obesidade, menor depressão e insatisfação corporal e maior autoestima do que as meninas. A depressão teve relação negativa com a autoestima, que também se associou negativamente com a insatisfação corporal.
19^34^	Examinar as associações da vitimização entre pares com sintomas internalizantes, competência social e desempenho acadêmico em uma amostra clínica de adolescentes com obesidade grave e se a autoestima e o apoio social afetam essas associações.	A autoestima mediou os efeitos da vitimização, sendo importante para a prevenção e intervenção tanto para a vitimização quanto para o mau ajustamento na obesidade grave entre adolescentes.
20^11^	Investigar o impacto de intervenções multicomponentes de controle de peso na autoestima em adolescentes com sobrepeso e obesos.	Adolescentes com sobrepeso e obesidade tendem a ter baixa autoestima. A meta-análise não encontrou melhoria significativa na autoestima, mesmo com perda de peso. Entretanto, as intervenções com maiores efeitos na autoestima também apresentaram maior efeito sobre a perda de peso.
21^35^	Avaliar a associação entre sobrepeso/obesidade e desempenho acadêmico geral futuro entre estudantes do ensino médio na Arábia Saudita.	Em um modelo de regressão logística múltipla ajustado para características sociodemográficas, estilo de vida relacionado ao estudo e autoestima, o sobrepeso/obesidade foi associado ao declínio do desempenho acadêmico.
22^36^	Investigar comportamentos alimentares disfuncionais e variáveis psicológicas tipicamente associadas a distúrbios alimentares, como baixa autoestima, perfeccionismo , vergonha, cuidado parental percebido e proteção em adolescentes obesos e com peso normal e examinar como os principais fatores de risco para Transtornos Alimentares interagem entre si, o que explica a vulnerabilidade à psicopatologia alimentar.	A autoestima não teve efeito direto no risco de Transtorno Alimentar. A vergonha corporal teve uma relação mais forte com a vulnerabilidade a problemas alimentares, entretanto a baixa autoestima teve uma influência direta na vergonha corporal que, por sua vez, afeta o risco de psicopatologia alimentar.
23^37^	Avaliar a autocompetência percebida e a baixa autoestima durante um programa intensivo de perda de peso em uma grande amostra de jovens obesos e relacioná-las ao peso inicial, gênero e perda de peso.	Os participantes que participaram do acampamento de verão obtiveram melhoras na autoestima global, reduzindo a baixa autoestima de 35% para 16%.
24^38^	Determinar os efeitos do treinamento aeróbico, treinamento de resistência e treinamento combinado no humor, imagem corporal e autoestima em adolescentes com obesidade.	A partir das intervenções de treinamento aeróbico, de resistência e combinado, registrou-se que todos os grupos melhoraram a imagem corporal e a autopercepção física. O treinamento de resistência mostrou maiores aumentos na autoestima global.
25^39^	Avaliar a influência do estado ponderal na QVRS^‡^ dos adolescentes.	Adolescentes com sobrepeso/obesidade apresentaram pior QVRS^‡^, menor resistência, menor capacidade para atividade física, menor participação familiar e maior influência dos pares. A autoestima foi menor entre participantes do sexo feminino.
26^40^	Analisar a influência sobre composição corporal autoestima em crianças 12 a 14 anos na cidade de Jaén, Espanha.	Valores estatisticamente menores de autoestima foram observados nas meninas em comparação aos meninos. O IMC^†^ mais alto também foi associado à baixa autoestima.
27^41^	Examinar diferenças de grupo relacionadas a provocações em aspectos de bem-estar psicológico, autopercepções e comportamentos.	Identificou-se que existe relação entre provocações sobre o peso e menor bem-estar psicológico. Adolescentes que relataram sofrer provocações apresentaram menor autoestima, mais sintomas depressivos e se percebiam como menos capazes fisicamente em comparação aos que não foram provocados.
28^42^	Examinar a satisfação dos adolescentes com sua imagem corporal correlacionada à sua autoestima.	A autoestima foi baixa entre os adolescentes com obesidade. A menor satisfação com a imagem corporal leva à baixa autoestima.
29^43^	Comparar os níveis de estresse, autoestima, enfrentamento, apoio social e humor depressivo entre adolescentes de minorias com peso normal e com sobrepeso/obesos.	Adolescentes com obesidade/acima do peso relataram autoestima significativamente menor do que aqueles com peso normal. A baixa autoestima também foi um preditor significativo do humor depressivo no grupo com sobrepeso/obesidade.
30^44^	Examinar correlatos baseados em peso, autoestima e depressão entre 119 adolescentes obesos afroamericanos em de 11 a 18 anos em busca de tratamento.	Os resultados indicaram uma associação entre provocações e diminuição da autoestima e aumento da depressão. A satisfação corporal mediou parcialmente a associação entre provocação e autoestima.
31^45^	Testar a hipótese de que a insatisfação corporal media a associação entre obesidade e comprometimento no bem-estar emocional em dois pontos nas vidas de uma amostra da população geral de adolescentes do sexo masculino e feminino.	Os participantes obesos tendem a ter menor autoestima e maior humor depressivo do que os participantes com peso normal. Além disso, os níveis absolutos de insatisfação corporal e humor depressivo permaneceram mais altos e os níveis de autoestima mais baixos para meninas, independentemente do peso corporal.
32^46^	Examinar características associadas à baixa autoestima em uma grande amostra nacional de jovens adolescentes.	Na análise multivariada, sexo feminino, raça hispânica, sobrepeso e obesidade, busca de sensações, rebeldia e tempo diário de televisão foram independentemente associados a baixa autoestima.
33^47^	Explorar tendências seculares entre 2000 e 2008 na autoestima física comparando coortes transversais de jovens adolescentes.	Houve uma Autoestima Global significativamente maior na coorte de 2008 em comparação a de 2000. Na coorte de 2008, no grupo de adolescentes com sobrepeso/obesos, a única diferença significativa foi uma Força Física percebida menor em meninas e uma Autoestima Global significativamente menor em meninos.
34^48^	Analisar as relações entre os valores de ansiedade, autoestima global e percepção de esforço durante exercício físico de intensidade crescente numa população de adolescentes obesas e não obesas.	Os valores de ansiedade registados antes do exercício estão significativamente correlacionados com os valores globais de autoestima no grupo com obesidade.
35^49^	Explorar o tipo e a frequência da psicopatologia em uma amostra clínica e não clínica de adolescentes obesos e em um grupo de controle de peso normal.	Houve uma maior proporção de psicopatologia (depressão, problemas comportamentais, baixa autoestima) entre adolescentes obesos clínicos do que entre adolescentes obesos não clínicos. As descobertas forneceram evidências de uma população de risco psicossocial em um subgrupo de obesos adolescentes.
36^50^	Investigar a mudança na imagem corporal, autoestima e preocupações em adolescentes obesos que frequentam um acampamento residencial para perda de peso.	Os participantes apresentavam maior insatisfação com o peso corporal e autoestima. A participação no acampamento de peso melhorou o estado psicológico dos adolescentes e os níveis globais de autoestima.
37^51^	Comparar autoestima, histórico social, competência social e acadêmica, problemas comportamentais e estilo de vida de adolescentes com obesidade.	A obesidade teve uma correlação não significativa com a autoestima autoavaliada. Embora essa relação fosse limitada, os participantes obesos demonstraram insatisfação com sua aparência física e habilidades atléticas, conforme esperado. Esses achados indicam que, apesar do excesso de peso, a percepção da autoestima dos adolescentes não foi fortemente influenciada pela obesidade.
38^52^	Estudar distúrbios alimentares e emocionais em meninas adolescentes com Diabetes Mellitus insulinodependente.	Meninas com obesidade e com ou sem Diabetes Mellitus apresentaram menores pontuações no escore total de autoestima.
39^53^	Investigar a autoestima de meninas adolescentes em relação ao peso.	Os resultados demonstram que a autoestima de meninas adolescentes está relacionada ao seu peso. À medida que o índice de obesidade aumentou, a autoestima diminuiu.

*QV = Qualidade de Vida; ^†^IMC = Índice de Massa Corporal; ^‡^QVRS = Qualidade de Vida Relacionada à Saúde

## Discussão

O mapeamento dos estudos permitiu identificar como se caracteriza a autoestima entre adolescentes com obesidade, seu impacto e intervenções possíveis para melhoria. A maioria dos estudos aponta que o diagnóstico de obesidade entre adolescentes está relacionado a uma menor autoestima^11^ ^,^ ^17^ ^-^ ^19^ ^,^ ^21^ ^,^ ^24^ ^,^ ^27^ ^,^ ^31^ ^,^ ^41^ ^-^ ^43^ ^,^ ^51^. A relação entre autoestima e o IMC foi examinada em diferentes investigações que revelaram uma correlação negativa e significativa, sugerindo que à medida que o IMC aumenta, a autoestima diminui^20^ ^,^ ^30^ ^,^ ^32^. Uma relação inversa também pode ser observada na literatura, indicando que apresentar baixa autoestima é um dos principais fatores que contribuem para obesidade e afeta seu tratamento^19^ ^,^ ^54^.

A obesidade reflete em uma menor autoestima pois pode influenciar de forma negativa na autopercepção durante a adolescência, principalmente entre o sexo feminino^18^ ^,^ ^22^ ^-^ ^23^ ^,^ ^28^ ^-^ ^29^ ^,^ ^33^ ^,^ ^39^ ^-^ ^40^ ^,^ ^45^ ^-^ ^46^ ^,^ ^52^ ^-^ ^53^. Essa diferença entre os gêneros é influenciada por fatores socioculturais, familiares e experiências individuais. A mídia, os padrões de beleza considerados aceitáveis, as expectativas sociais e as pressões acadêmicas podem impactar a autoestima de forma distinta, o que contribui para a formação de identidades e autopercepções únicas entre os sexos^55^.

A baixa autoestima está associada à insatisfação com a vida, problemas familiares, menor apoio social percebido, saúde emocional e bem-estar negativos e transtornos mentais, como depressão e ansiedade, considerada um preditor de maiores taxas de suicídio nesse grupo^23^ ^,^ ^32^ ^,^ ^41^ ^,^ ^43^ ^,^ ^49^ ^,^ ^56^ ^-^ ^57^. Outro ponto é que sintomas de ansiedade e depressão, comuns na obesidade, contribuem na diminuição dos níveis de autoestima^19^ ^-^ ^20^ ^,^ ^45^. Destaca-se que a influência da imagem corporal, aceitação social e deterioração do humor é maior no sexo feminino, fato que o torna mais vulnerável aos transtornos psicossociais^58^ ^-^ ^59^.

A baixa autoestima entre adolescentes com obesidade também pode ser considerada um fator de risco para manifestações clínicas de Transtornos Alimentares, principalmente entre mulheres, pois a busca pelo corpo considerado “ideal” pode levar à adoção de comportamentos alimentares inadequados^22^. A baixa autoestima também influencia diretamente na vergonha corporal, que por sua vez, é um preditor de risco para psicopatologias alimentares^36^.

O preconceito contra a obesidade e as provocações quanto ao peso são comuns no meio social^41^. Há evidências de que ser obeso é identificado como uma das condições menos aceitáveis socialmente e possivelmente mais estigmatizante, principalmente na infância e adolescência^60^. Níveis de autoestima de crianças com obesidade diminuem com a idade e as dificuldades psicossociais tornam-se mais pronunciadas à medida que crescem e entram na adolescência, pois adolescentes com obesidade são mais propensos a sofrer provocações de seus pares, o que pode levar à marginalização social^24^ ^,^ ^61^.

A exposição a provocações relacionadas ao peso, intimidação ou vitimização também contribuem para o desenvolvimento de problemas psicológicos e emocionais, que levam a um baixo desempenho escolar^27^ ^,^ ^34^ ^-^ ^35^. Também foi relatado que o desempenho escolar é influenciado pelo excesso de peso ou o absenteísmo dos estudantes com obesidade devido aos problemas de saúde que podem enfrentar^24^. Diferentes explicações foram sugeridas para a associação negativa entre IMC e desempenho acadêmico e uma delas inclui o impacto negativo na autoestima^35^.

Apesar desta revisão destacar a relação negativa entre obesidade e autoestima, esta última, quando avaliada positivamente está relacionada a melhores condições de bem-estar e saúde mental, autopercepções positivas, realizações acadêmicas e persistência^31^. Isso reforça a importância da prevenção e controle da obesidade ainda na infância, bem como estratégias de aconselhamento para ajudar no controle do excesso de peso entre adolescentes^46^.

Estudos mostram que adolescentes com melhor desempenho escolar e com participação em esportes coletivos apresentam menor risco de baixa autoestima, destacando uma oportunidade de incentivo para o desenvolvimento de programas esportivos escolares e comunitários que colaborem com a prevenção da obesidade e promoção de melhor autoestima^21^ ^,^ ^31^ ^,^ ^46^. Indivíduos com maior autoestima também se sentem mais motivados a praticar atividade física^18^ ^,^ ^21^ ^,^ ^62^.

Práticas esportivas, como o Muay Thai, foram aplicadas como intervenção para melhoria da autoestima entre adolescentes com obesidade^16^. Essas práticas trazem contribuições não apenas para a saúde física e psíquica, mas também favorecem as competências sociais^63^. Um estudo que ofereceu um programa de Karatê com duração de mais de dez semanas, para crianças e adolescentes com epilepsia, mostrou melhorias na autoestima dos praticantes^64^. Outro analisou a autoestima, autoconfiança e ansiedade cognitiva e somática em adultos praticantes e não praticantes de Jiu-Jitsu, e observou que os praticantes apresentaram maiores níveis de autoestima e autoconfiança^65^.

Os treinamentos de aeróbica e de resistência e a combinação de ambos mostraram efeitos positivos na saúde psicológica de adolescentes com obesidade. Verificou-se que a prática, em uma frequência de pelo menos seis meses, resultou em melhorias na autoestima global e física. Fatores associados ao efeito do treinamento são sugestivos ao potencial de remodelação do corpo e melhorias no tamanho corporal, forma e tônus muscular, além da redução do IMC^38^.

As intervenções realizadas em acampamentos apresentaram potenciais efeitos na autoestima global dos adolescentes, isso se deve à combinação de atividades desenvolvidas, com ênfase nos aspectos nutricionais, físicos e emocionais, preditor associado a melhoras no nível de autoestima também foi a redução do peso. Em um dos estudos que utilizou a intervenção na ambientação de acampamento, os participantes tiveram em média uma redução do peso em 5,6 kg^50^.

Ademais, intervenções com ambientação de acampamento também obtiveram melhoras significativas na autoestima global dos adolescentes com obesidade, mesmo quando não havia a redução significativa de peso aparente, o que pode estar associado à autoeficácia, como a prática de exercício regular e um ambiente de apoio que podem favorecer o desenvolvimento de competências e maiores interações sociais, estabelecendo novas amizades ou estando em um ambiente menos vulnerável às experiências associadas ao estigma e à vitimização^37^.

As intervenções de cunho educacional apresentam múltiplos benefícios, isso ocorre devido à natureza dessas intervenções, de explorar diferentes habilidades como aprendizagem, comportamentos e atitudes, possibilitando modificações e reflexões acerca da autoestima, afastando os estigmas, provocações dos colegas e padrões estéticos socialmente definidos, aproximando-os de crenças e valores congruentes à autovalorização e autoconfiança em si mesmo. Cabe ressaltar que o período da intervenção é fundamental para garantir os potenciais efeitos. A aplicação de um programa educacional no período de 12 meses foi capaz de identificar melhorias na satisfação corporal, mudanças de atitudes e percepção relacionados à autoestima^40^.

Reforça-se também a importância das intervenções baseadas no familismo, na qual, em estudo realizado com adolescentes latinos com obesidade, a relação familiar representou um fator de proteção para a autoestima e, consequentemente, associado ao bem-estar geral. Famílias estruturadas e estáveis podem proporcionar ao adolescente uma visão mais abrangente sobre suas qualidades e valores que vão além do peso e aparência física, fornecendo uma rede apoio e competências para desenvolver relacionamentos interpessoais com mais facilidade e engajar-se em atividades alinhadas aos seus objetivos^25^.

O período da infância desempenha um papel importante na formação da autoestima de crianças e adolescentes. Cuidadores sensíveis e solidários contribuem para a formação do apego em crianças. À medida que recebem feedbacks positivos, formam um autoconceito positivo, o que as tornam mais seguras e com melhor autoestima^26^. A autoestima formada durante a infância pode continuar durante a adolescência. Assim, a família é um dos fatores mais importantes que influenciam a autoestima dos adolescentes, especialmente entre as meninas^66^.

Uma revisão sistemática identificada apontou que as intervenções com multicomponentes tais como alimentação, educação e atividade física mostraram benefícios na melhora da autoestima, apesar da relação entre autoestima e obesidade não estar totalmente esclarecida. A adoção dessas estratégias, com foco na autoestima do adolescente, pode ser eficiente para redução do IMC e melhora na qualidade de vida desses indivíduos. Quanto aos instrumentos utilizados para avaliação da autoestima, foram identificados dois diferentes tipos de instrumentos, dentre eles: Escala de Autoestima de Rosenberg e o Perfil de Autopercepção de Harter para Crianças/Adolescentes^11^.

Tais dados corroboram com os encontrados nesta revisão, em que a avaliação da autoestima foi realizada de forma quantitativa, apoiando-se no uso de instrumentos validados, sendo o mais utilizado a Escala de Autoestima de Rosenberg^11^ ^,^ ^16^ ^-^ ^21^ ^,^ ^25^ ^-^ ^26^ ^,^ ^30^ ^,^ ^32^ ^-^ ^34^ ^,^ ^40^ ^-^ ^41^ ^,^ ^45^ ^,^ ^49^ ^,^ ^53^. As versões validadas e adaptadas para os idiomas português^33^ e espanhol^17^ também foram utilizadas. Em seguida, tem-se o Inventário de Autoestima Coopersmith, que avalia a autoestima em diferentes áreas, familiar, acadêmica, social e geral^27^ ^,^ ^41^ ^,^ ^44^ ^,^ ^52^; e a escala Perfil de Autopercepção de Harter para Crianças/Adolescentes, que avalia de forma mais detalhada os aspectos da vida dos indivíduos quanto à dimensão global da autoestima em diferentes dimensões ou domínio da vida^11^ ^,^ ^38^.

A variedade de instrumentos identificados na síntese de resultados realça a complexidade da avaliação da autoestima, bem como a investigação desse conceito em diferentes aspectos do indivíduo, seja em abordagens mais abrangentes como autoestima global ou em componentes específicos dentro da autoestima e como estes se relacionam e impactam a nível psicológico e social na vida das pessoas, incluindo adolescentes.

Os estudos transversais foram amplamente utilizados para avaliar a autoestima entre os adolescentes e correlacioná-la a outras variáveis^17^ ^-^ ^18^ ^,^ ^21^ ^,^ ^23^ ^-^ ^33^ ^,^ ^39^ ^-^ ^40^ ^,^ ^42^ ^,^ ^44^ ^,^ ^52^ ^-^ ^53^. Esse tipo de estudo é útil para determinar a prevalência de uma condição de interesse sobre uma população em um determinado período de tempo, identificar associações, gerar hipóteses para investigações futuras e contribuir no planejamento de intervenções. Entretanto, apresenta fragilidades, como não permitir estabelecer relações de causalidade e análise da evolução de um determinado constructo ao longo do tempo. Ademais, é suscetível a vieses de seleção pois a amostra da população de interesse pode não ser representativa, comprometendo a generalização dos resultados^67^.

Ademais, o papel das equipes de saúde é fundamental para o rastreio e identificação precoce de adolescentes com altos níveis de estresse e baixa autoestima. Para isso, esses profissionais podem atuar através de programas de promoção da saúde da escola, tendo em vista que este é um ambiente de acesso aos adolescentes, podendo utilizar de instrumentos confiáveis e validados, exames de saúde mental, além de projetos de aconselhamento e integração escolar^23^ ^,^ ^43^.

Nesse ponto, destaca-se que a multidisciplinaridade da equipe é algo essencial para construção de uma rede de apoio aos adolescentes com obesidade, onde cada profissional desempenha um papel-chave. A equipe pode ser constituída por pais, professores, assistentes sociais, conselheiros e psicólogos estabelecendo uma força-tarefa capaz de promover um ambiente de apoio que permita aos adolescentes desenvolverem habilidades, competências e confiança para explorar questões de saúde mental, obesidade e autoestima^22^ ^-^ ^23^ ^,^ ^43^ ^,^ ^45^.

Vale ressaltar que os enfermeiros estão em uma posição favorável, pois, no que tange à promoção da saúde na escola, é esse profissional que estabelece uma comunicação prévia com professores, pais, alunos e outros funcionários. Ao observar e conversar com os adolescentes é possível identificar sinais de cansaço, humor depressivo, desânimo, que podem alertar os enfermeiros sobre a necessidade de encaminhamento desse adolescente para uma avaliação mais completa com outros profissionais de saúde mental^36^ ^,^ ^41^ ^,^ ^43^.

Outro ponto importante é que os profissionais de saúde precisam estar cientes dos efeitos da baixa autoestima na saúde mental dos adolescentes com obesidade, tendo em vista que as intervenções precoces e outras abordagens terapêuticas geralmente têm como foco o peso corporal, entretanto também devem estar alinhadas à autoestima que influencia tanto no bem-estar do adolescente como na presença de sofrimento psíquico^23^ ^,^ ^45^.

Como limitação desta revisão, destaca-se a abordagem utilizada pelos estudos incluídos. Apesar do uso de instrumentos validados para mensuração da autoestima entre adolescentes com obesidade, é importante destacar que a autoestima é um conceito complexo, influenciado por diferentes variáveis e contextos, os quais devem ser considerados para uma avaliação mais abrangente. Outra limitação refere-se ao fato de que em alguns estudos a autoestima foi avaliada apenas no momento da aplicação da intervenção, sendo necessário realizar avaliações pós-intervenção, já que a autoestima pode variar ao longo do tempo e ser afetada por múltiplos fatores.

Os achados deste estudo contribuem para avanços na área da Enfermagem ao aprofundar a compreensão sobre a autoestima de adolescentes com obesidade. Além disso, a identificação dos fatores associados à baixa autoestima e as vulnerabilidades presentes nesse grupo, principalmente a influência dos padrões sociais impostos ao sexo feminino requer preparo e olhar atento deste profissional. Ainda, destaca-se o papel do Enfermeiro de educador em saúde ao orientar potenciais intervenções para a promoção da autoestima, considerando que melhorias nesse aspecto impactam diretamente no bem-estar. O estudo colabora com evidências para a prática clínica e estratégias de cuidado holístico e multiprofissional mais eficazes e sensíveis ao contexto psicossocial do adolescente.

## Conclusão

A adolescência é um período de mudanças no qual ocorrem oscilações psicológicas, seja atreladas ao humor, seja às emoções ou à estima. Quando associadas a problemas de saúde, essas alterações podem ocorrer de forma mais intensa, resultando em impactos negativos sobre a saúde mental.

Os resultados de alguns estudos incluídos na revisão indicam que a obesidade em adolescentes favorece a baixa autoestima, sendo que, em alguns casos, foi considerada um preditor para o aumento do IMC. Diante disso, destaca-se que a baixa autoestima é frequentemente identificada entre o público feminino, fator que pode ter relação com as pressões sociais e influências dos pares e das mídias quanto aos padrões de beleza impostos e aceitos pela sociedade.

A baixa autoestima afeta diferentes dimensões da vida do adolescente com obesidade, tais como física, mental e social. Além disso, amplia as vulnerabilidades a Transtornos Mentais e Alimentares, contribuindo, em casos mais graves, para o risco de comportamentos suicidas. 

Destaca-se que intervenções a nível escolar e no cotidiano dos adolescentes, pautadas na reeducação de hábitos sedentários, atividade física e esportivas, bem como na ênfase sobre os aspectos emocionais e no desenvolvimento de competências e habilidades apresentam-se como potenciais para a melhoria da autoestima. O papel da família é essencial, oferecendo apoio e servindo como exemplo de valorização da singularidade do adolescente, percebendo-o além do diagnóstico de obesidade, considerando suas necessidades, gostos, qualidades e expectativas.

Sugere-se a realização novos estudos, principalmente de abordagem qualitativa, que explorem com maior profundidade e complexidade a percepção de adolescentes com obesidade em relação à autoestima, considerando as dimensões biopsicossociais desse público. O investimento em pesquisas na área favorece o desenvolvimento de estratégias de intervenção que busquem a promoção da saúde e do bem-estar dos adolescentes. 

## Data Availability

Todos os dados gerados ou analisados durante este estudo estão incluídos neste artigo publicado.

# Translation: Autoestima en adolescentes con obesidad: revisión de alcance^*^

**DOI:** 10.1590/1518-8345.7936.4887

**Authors:** Chrisllayne Oliveira da Silva, Nanielle Silva Barbosa, Ana Larissa Gomes Machado, Ítalo Arão Pereira Ribeiro, Ana Maria Ribeiro dos Santos, Márcia Astrês Fernandes

**Affiliations:** 1 Universidade Federal do Piauí, Teresina, PI, Brasil.; 2 Becaria de la Coordenação de Aperfeiçoamento de Pessoal de Nível Superior (CAPES), Brasil.; 3 Universidade Federal de Mato Grosso do Sul, Coxim, MS, Brasil.

**Keywords:** Adolescente, Autoimagen, Obesidad, Bienestar Psicológico, Enfermería, Revisión

## Abstract

**Objetivo:**

mapear las evidencias científicas acerca de la evaluación, la percepción y las intervenciones dirigidas a la autoestima en adolescentes con obesidad.

**Método:**

revisión de alcance, cuyo protocolo fue registrado en la plataforma *Open Science Framework* con el identificador 10.17605/OSF.IO/C6W72. Se incluyeron estudios primarios y secundarios, disponibles en texto completo, sin restricción temporal ni de idioma, de ocho bases de datos y literatura gris. Se excluyeron cartas al editor, estudios en fase de protocolo o aún sin resultados.

**Resultados:**

se seleccionaron 39 estudios para el análisis. La síntesis de evidencias en los adolescentes con obesidad investigados indicó baja autoestima, la cual se asoció con el sexo femenino, la vergüenza corporal, la exposición a la victimización por pares, síntomas depresivos y bajo rendimiento escolar. Las intervenciones con potenciales resultados en la mejora de la autoestima se relacionaron con la reducción de peso, como prácticas deportivas, programas educativos y apoyo familiar.

**Conclusión:**

existe una relación compleja entre la obesidad en adolescentes y la baja autoestima, lo que hace fundamental el establecimiento de buenas prácticas alimentarias y actividades físicas regulares, además del apoyo emocional a los adolescentes considerando las dimensiones biopsicosociales.

## Destacados

**(1)** Los adolescentes con obesidad presentan menor autoestima. **(2)** La baja autoestima afecta el bienestar físico y mental y las relaciones sociales. **(3)** El apoyo familiar es uno de los factores que influyen en la formación de la autoestima. **(4)** Las buenas prácticas alimentarias y las actividades físicas regulares favorecen la autoestima.

## Introducción

La adolescencia se considera generalmente una fase de transición, aunque el individuo no es visto como niño, aún no se considera un adulto. Es precisamente en este período cuando el desarrollo humano ocurre de forma acelerada, ya sea en aspectos físicos, cognitivos, sociales, sexuales y emocionales^1^. Estos cambios intensos pueden contribuir a situaciones de riesgo para la salud de este adolescente y al desarrollo de Enfermedades Crónicas No Transmisibles (ECNT) como la obesidad, que es la acumulación excesiva o anormal de grasa corporal^1^ ^-^ ^3^.

Los adolescentes con obesidad se enfrentan constantemente con factores externos, como dificultad de aceptación e inclusión en grupos, estigma social y *bullying*, que pueden resultar en bajos niveles de autoestima, conduciendo a un cuadro de vulnerabilidad para trastornos alimentarios, ansiedad, depresión y aislamiento social^4^ ^-^ ^5^.

Ante lo expuesto, la autoestima de los adolescentes debe considerarse una variable relevante para evaluar la salud de estos individuos, considerando que está vinculada a la construcción de la identidad y a la adaptación al entorno, funcionando como uno de los reguladores del bienestar^6^ ^-^ ^7^.

En una investigación de base nacional con estudiantes brasileños de 12 a 17 años, tres de cada diez estudiantes presentan insatisfacción con la imagen corporal, mientras que los adolescentes con obesidad son propensos a la baja autoestima y a la negligencia en relación con la autopercepción asociada al exceso de peso y al riesgo de comorbilidades^2^.

En este sentido, la autoestima, definida como una autoevaluación sobre uno mismo, sobre las propias actitudes y la relación con los demás, influye en cómo el individuo reacciona ante los cambios internos y externos, además de atribuir valores y sentimientos favorables o desfavorables que, cuando son negativos, impactan en la salud del adolescente. Por otro lado, en niveles satisfactorios, puede influir en la adopción de actitudes de promoción de la salud y protección frente a comportamientos perjudiciales^6^ ^,^ ^8^ ^-^ ^9^.

A partir de una búsqueda previa en la literatura, se identificaron dos estudios acerca de la relación entre obesidad y autoestima: una revisión de alcance con el propósito de mapear los instrumentos existentes para medir la autoestima en niños y adolescentes y una revisión sistemática que se propuso evaluar si intervenciones multicomponente tendrían impacto en la autoestima de los adolescentes^10^ ^-^ ^11^. Así, el estudio se diferencia de los demás al abordar aspectos referentes a la percepción y a los conceptos de los adolescentes con obesidad frente a la autoestima y cómo esto impacta en la vida cotidiana, además de comprender la relación entre obesidad y autoestima como un factor de enfermar o protector.

A pesar de la cantidad significativa de estudios acerca de la obesidad en la adolescencia en diferentes bases de datos, aún existe una laguna en relación con la autoestima, que es un factor importante en el desarrollo personal y en el mantenimiento de las condiciones de salud.

De este modo, comprender la percepción de los adolescentes con obesidad puede aportar evidencias relevantes sobre las influencias en la autoimagen, considerando que la obesidad puede ser un factor de riesgo para un autoconcepto negativo, pudiendo agravar una baja autoestima. Además, la información obtenida puede contribuir al desarrollo de futuras investigaciones que puedan orientar intervenciones con énfasis en las complejidades y en la mitigación del estigma asociado a la obesidad y sus impactos en la salud mental.

Este estudio tuvo como objetivo mapear las evidencias científicas acerca de la evaluación, la percepción y las intervenciones dirigidas a la autoestima en adolescentes con obesidad.

## Método

### Diseño del estudio

Estudio de revisión de la literatura, del tipo revisión de alcance, realizado de acuerdo con las etapas recomendadas por el JBI: título y preguntas de revisión, criterios de inclusión, estrategia de búsqueda, cribado y selección de evidencias, extracción y análisis de datos y presentación de los resultados; y descrito utilizando las directrices del *Preferred Reporting Items for Systematic Reviews and Meta-Analyses extension for Scoping Review* (PRISMA-ScR)^12^ ^-^ ^13^.

El protocolo de la revisión fue registrado en *Open Science Framework* (OSF) con el identificador DOI 10.17605/OSF.IO/C6W72. Antes de la elaboración del protocolo, los autores verificaron en la literatura la existencia de otras revisiones con tema y objetivos similares a los de esta revisión. Se encontraron dos revisiones; sin embargo, estuvieron dirigidas a los instrumentos de evaluación de la autoestima y a intervenciones específicas, diferenciándose de los objetivos del presente estudio^10^ ^-^ ^11^.

### Escenario

Las bases de datos consultadas fueron: Literatura Latinoamericana y del Caribe en Ciencias de la Salud (LILACS), Base de Datos de Enfermería (BDENF), Índice Bibliográfico Español en Ciencias de la Salud (IBECS), *Index* Psicología (INDEXPSI), vía Biblioteca Virtual en Salud (BVS), *Medical Literature Analysis and Retrieval System Online* (MEDLINE) vía PubMed, *Web of Science*, *Scopus* y APA PsycInfo^®^.

La literatura gris se recuperó en fuentes como la *Biblioteca Digital Brasileira de Teses e Dissertações* (BDTD), el *Catálogo de Teses e Dissertações* (CTD) de la *Coordenação de Aperfeiçoamento de Pessoal de Nível Superior* (CAPES) y *Google Scholar* (en las 10 primeras páginas).

### Período

La búsqueda se realizó entre diciembre de 2024 y enero de 2025.

### Población

Se utilizó el acrónimo PCC, siendo la Población los adolescentes, caracterizados como individuos con edades entre 10 y 19 años, conforme a la definición de la Organización Mundial de la Salud (OMS). El Concepto adoptado fue la autoestima y el Contexto de interés fue la obesidad. La pregunta de revisión fue: ¿cuáles son las evidencias disponibles acerca de la evaluación, la percepción y las intervenciones dirigidas a la autoestima en adolescentes con obesidad? 

### Criterios de selección

Se incluyeron estudios primarios y secundarios sobre autoestima en adolescentes con obesidad, tanto estudios que consideraron estas dos variables como principales como aquellos que analizaron diferentes variables, pero que verificaron la autoestima en adolescentes con la comorbilidad, primarios y secundarios, disponibles en texto completo, sin restricción temporal ni de idioma. Se excluyeron cartas al editor y estudios en fase de protocolo o aún sin resultados. 

### Definición de la muestra

Se realizó una búsqueda inicial sobre la temática de interés en la literatura, con el apoyo de una bibliotecaria. A partir de los estudios recuperados, se llevó a cabo un análisis de los términos contenidos en el título, resumen, texto y descriptores/palabras clave a fin de seleccionarlos en los Descriptores en Ciencias de la Salud (DeCS) y *Medical Subject Headings* (MeSH). Estos términos se combinaron entre sí, de modo de garantizar el mayor retorno en la cantidad de estudios, estructurando las estrategias de búsqueda específicas para cada base de datos, conforme a la Figura 1. Las estrategias fueron probadas por cada una de las dos revisoras y por la bibliotecaria, de manera independiente, a fin de garantizar el mismo resultado en todas las búsquedas.

**Figura 1:** Estrategia de búsqueda en bases de datos y literatura gris. Teresina, PI, Brasil, 2024

Base de datos	Estrategia de búsqueda
LILACS*/BDENF^†^/IBECS^‡^/INDEXPSI^§^ (BVS^ǁ^)	((mh:(adolescente )) OR (adolescentes) AND (adolescência)) AND ((mh:(autoimagem)) OR (autoestima)) AND ((mh:(obesidade)) OR (obesidade)) AND db:(“LILACS*” OR “IBECS^‡^” OR “BDENF^†^” OR “INDEXPSI^§^”) AND instance:”regional”
MEDLINE^¶^ (PubMed)	(((“self concept”[MeSH Terms]) OR (“self esteem”[All Fields])) AND (((“adolescent”[MeSH Terms]) OR (“adolescents”[All Fields])) OR (“adolescence”[All Fields]))) AND ((Obesity[MeSH Terms]) OR (“obesity”[All Fields]))
*Web of Science*	((ALL=(Adolescent) OR ALL=(Adolescents) OR ALL=(Adolescence)) AND ALL=(Obesity)) AND (ALL=(‘’Self Concept’’) OR ALL=(‘’Self Esteem’’))
*Scopus*	( ( TITLE-ABS-KEY ( adolescent ) OR TITLE-ABS-KEY ( adolescents ) OR TITLE-ABS-KEY ( adolescence ) ) ) AND ( TITLE-ABS-KEY ( obesity ) ) AND ( ( TITLE-ABS-KEY ( ‘’self concept’’ ) OR TITLE-ABS-KEY ( self esteem ) ) )
APA** PsycInfo^®^	((Any Field: (Adolescent)) OR (Any Field: (Adolescents)) OR (Any Field: (Adolescence))) AND ((Any Field: (Self concept)) OR (Any Field: (Self Esteem))) AND ((Any Field: (Obesity)))
**Literatura gris**	**Estrategia de búsqueda**
BDTD^††^	(Adolescentes OR adolescente OR adolescência) AND (Autoimagem OR Autoestima) AND (Obesidade)
CTD-CAPES^‡‡^	(Adolescentes OR adolescente OR adolescência) AND (Autoimagem OR Autoestima) AND (Obesidade)
*Google Scholar*	Adolescents AND ‘’Self esteem’’ AND Obesity

*LILACS = Literatura Latinoamericana y del Caribe en Ciencias de la Salud; ^†^BDENF = Base de Datos de Enfermería; ^‡^IBECS = Índice Bibliográfico Español en Ciencias de la Salud; ^§^INDEXPSI = Index Psicología; ^ǁ^BVS = Biblioteca Virtual en Salud; ^¶^MEDLINE = *Medical Literature Analysis and Retrieval System Online*; ^**^APA = *American Psychological Association*; ^††^BDTD = *Biblioteca Digital Brasileira de Teses e Dissertações*; ^‡‡^CTD-CAPES = *Catálogo de Teses e Dissertações da Coordenação de Aperfeiçoamento de Pessoal de Nível Superior*

### Recolección de datos

La búsqueda, la selección y el análisis se realizaron de forma independiente, a ciegas, por dos revisoras, autoras del estudio, una con titulación de Magíster en Ciencias y Salud y con conocimiento en el área de Salud del Niño y del Adolescente, y la otra Magíster en Enfermería y con conocimiento en el área de Salud Mental, ambas con experiencia en la elaboración y publicación de revisiones. Las divergencias en cuanto a la inclusión o no de determinado texto, tras la discusión entre las dos primeras revisoras, fueron resueltas por una tercera revisora, enfermera Doctora en Enfermería con conocimiento en el área de Salud Colectiva.

Las citas identificadas se importaron al gestor de referencias Rayyan^®^, desarrollado por el *Qatar Computing Research Institute* (QCRI) para la eliminación de duplicados, la selección y el cribado.

La primera fase de selección comprendió la lectura de títulos y resúmenes. Los estudios que cumplieron los criterios de inclusión se analizaron en una segunda fase, mediante la lectura de los manuscritos en texto completo.

Los motivos de exclusión de los estudios se registraron y se informaron en un diagrama de flujo^14^.

### Variables del estudio

Las variables extraídas de los estudios fueron: autoría, país de origen, idioma, año de publicación, objetivo, tipo de estudio, muestra, lugar de la recolección de datos, instrumento de evaluación de la autoestima, principales resultados y Nivel de Evidencia^15^.

### Instrumento de recolección de datos

La información se mapeó conforme a las recomendaciones del JBI para la caracterización de las fuentes de evidencia, registradas y organizadas en formato de cuadro sinóptico elaborado en *Microsoft Office Excel* 2024.

### Procesamiento y análisis de datos 

El análisis de los datos se realizó de forma descriptiva, cuantitativa y cualitativa. La síntesis de los resultados se presentó de forma narrativa y mediante figuras. 

### Aspectos éticos

El estudio utilizó datos secundarios; por lo tanto, no hubo evaluación por el Comité de Ética en Investigación (CEP).

## Resultados

Se identificaron 6.860 estudios en las bases de datos, de los cuales 2.474 se excluyeron por duplicado y 4.386 pasaron a la lectura del título y del resumen. Tras la primera lectura, se seleccionaron 50 para lectura en texto completo, de los cuales 36 se incluyeron en la muestra final. A partir de la literatura gris, se incluyeron tres estudios. Así, 39 estudios conformaron la muestra final de la revisión, conforme se presenta en la Figura 2.

La descripción de los estudios según el país de origen, año de publicación, idioma, tipo de estudio, Nivel de Evidencia, muestra, lugar e instrumento de evaluación de la autoestima se encuentra en la Figura 3.

**Figura 2:** Diagrama de flujo de selección de los estudios. Teresina, PI, Brasil, 2024

*BVS = Biblioteca Virtual en Salud; ^†^MEDLINE = *Medical Literature Analysis and Retrieval System Online*; ^‡^APA = *American Psychological Association*; ^§^BDTD = *Biblioteca Digital Brasileira de Teses e Dissertações*; ^ǁ^CTD-CAPES = *Catálogo de Teses e Dissertações da Coordenação de Aperfeiçoamento de Pessoal de Nível Superior*

**Figura 3:** Descripción de los estudios seleccionados en cuanto al país de origen, año de publicación, idioma, tipo de estudio, Nivel de Evidencia, muestra, lugar e instrumento de evaluación de la autoestima. Teresina, PI, Brasil, 2024

Orden	País de origen/año de publicación/idioma	Método
01^16^	Brasil/2024/Inglés	Tipo de estudio/Nivel de Evidencia: ensayo clínico aleatorizado controlado/II Muestra: 40 adolescentes recibieron la intervención y 13 participaron en el grupo control, con edades entre 10 y 17 años. Lugar: centro de estudios. Instrumento: Escala de Autoestima de Rosenberg.
02^17^	Chile/2024/Español	Tipo de estudio/Nivel de Evidencia: transversal/VI Muestra: 397 adolescentes en el rango de edad de 10 a 19 años. Lugar: escuelas. Instrumento: Escala de Autoestima de Rosenberg.
03^18^	Marruecos/2024/Inglés	Tipo de estudio/Nivel de Evidencia: transversal/VI Muestra: 410 adolescentes con edades entre 12 y 17 años. Lugar: escuelas. Instrumento: Escala de Autoestima de Rosenberg.
04^19^	Turquía/2023/Inglés	Tipo de estudio/Nivel de Evidencia: casos y controles/IV Muestra: 50 niños con diagnóstico de obesidad y 48 participantes en el grupo control, con edades entre 11 y 17 años. Lugar: universidad. Instrumento: Escala de Autoestima de Rosenberg.
05^20^	Reino Unido/2023/Inglés	Tipo de estudio/Nivel de Evidencia: cohorte prospectiva/IV Muestra: 18.818 participantes de 11 a 17 años. Lugar: no especificado. Instrumento: Escala de Autoestima de Rosenberg.
06^21^	Reino Unido/2022/Inglés	Tipo de estudio/Nivel de Evidencia: transversal/VI Muestra: 9046 adolescentes de 11 y 14 años Lugar: domicilios. Instrumento: Escala de Autoestima de Rosenberg.
07^22^	China/2021/Inglés	Tipo de estudio/Nivel de Evidencia: casos y controles/IV Muestra: 128 estudiantes con obesidad y 128 con peso normal. Lugar: escuela. Instrumento: Escala de Estereotipo Corporal para Adolescentes y Adultos.
08^23^	Estados Unidos de América/2021/Inglés	Tipo de estudio/Nivel de Evidencia: transversal/VI Muestra: 6.748 adolescentes. Lugar: escuelas. Instrumento: Versión modificada del Inventario de Autoestima de Rosenberg.
09^24^	Grecia/2020/Inglés	Tipo de estudio/Nivel de Evidencia: transversal/VI Muestra: 528 adolescentes con edades entre 10 y 12 años. Lugar: escuela. Instrumento: Perfil de Autopercepción para Niños.
10^25^	Estados Unidos de América/2020/Inglés	Tipo de estudio/Nivel de Evidencia: transversal/VI Muestra: 160 adolescentes de 14 a 16 años. Lugar: centros comunitarios. Instrumento: Escala de Autoestima de Rosenberg.
11^26^	Irán/2020/Inglés	Tipo de estudio/Nivel de Evidencia: transversal/VI Muestra: 173 adolescentes con edades entre 12 y 16 años. Lugar: centro de estudios. Instrumento: Escala de Autoestima de Rosenberg.
12^27^	Chile/2019/Español	Tipo de estudio/Nivel de Evidencia: transversal/VI Muestra: 419 adolescentes entre 14 y 17 años. Lugar: escuela. Instrumento: Inventario de Autoestima de Coopersmith.
13^28^	Egipto/2019/Inglés	Tipo de estudio/Nivel de Evidencia: transversal/VI Muestra: 533 adolescentes de 10 a 19 años. Lugar: escuela. Instrumento: Escala de Autoestima de Rosenberg.
14^29^	Chile/2018/Inglés	Tipo de estudio/Nivel de Evidencia: transversal/VI Muestra: 712 escolares de 10 a 13 años. Lugar: escuela. Instrumento: TAE-student*.
15^30^	España/2018/Español	Tipo de estudio/Nivel de Evidencia: transversal/VI Muestra: 364 escolares entre 11 y 13 años. Lugar: centros educativos públicos. Instrumento: TAE-student*.
16^31^	Chile/2018/Inglés	Tipo de estudio/Nivel de Evidencia: transversal/VI Muestra: 656 adolescentes entre 11 y 14 años. Lugar: escuela. Instrumento: TAE-student*.
17^32^	India/2017/Inglés	Tipo de estudio/Nivel de Evidencia: transversal/VI Muestra: 150 adolescentes con una media de edad de 14,5 años. Lugar: escuela. Instrumento: Escala de Autoestima de Rosenberg.
18^33^	Brasil/2017/Portugués	Tipo de estudio/Nivel de Evidencia: transversal/VI Muestra: 418 adolescentes de 14 y 18 años. Lugar: escuela. Instrumento: Escala de Autoestima de Rosenberg.
19^34^	Estados Unidos de América/2017/Inglés	Tipo de estudio/Nivel de Evidencia: cohorte prospectiva/IV Muestra: 139 adolescentes con edades entre 13 y 18 años. Lugar: hospital. Instrumento: Perfil de Autopercepción para Adolescentes.
20^11^	Australia/2017/Inglés	Tipo de estudio/Nivel de Evidencia: revisión sistemática y metanálisis/I Muestra: trece estudios con 1.157 adolescentes con sobrepeso u obesidad, con edades entre 10 y 19 años. Lugar: escuela y domicilios. Instrumento: Escala de Autoestima de Rosenberg; Perfil de Autopercepción de Harter para Niños; Autoestima Global de Marsh.
21^35^	Arabia Saudita/2016/Árabe	Tipo de estudio/Nivel de Evidencia: cohorte retrospectiva/IV Muestra: 257 adolescentes con una media de edad de 18 años. Lugar: escuela. Instrumento: Escala de Autoestima de Rosenberg.
22^36^	Italia/2016/Inglés	Tipo de estudio/Nivel de Evidencia: casos y controles/IV Muestra: 111 estudiantes con obesidad y 111 con peso normal, en el rango de edad de 13 a 19 años. Lugar: escuela. Instrumento: Escala de Autoestima de Rosenberg.
23^37^	Reino Unido/2016/Inglés	Tipo de estudio/Nivel de Evidencia: ensayo clínico aleatorizado controlado/II Muestra: 303 jóvenes con edades entre 11 y 17 años. Lugar: domicilios. Instrumento: Perfil de Autopercepción para Niños.
24^38^	Canadá/2015/Inglés	Tipo de estudio/Nivel de Evidencia: ensayo clínico aleatorizado controlado/II Muestra: 304 participantes de 14 a 18 años. Lugar: instalaciones comunitarias. Instrumento: Perfil de Autopercepción Física para Niños; Subescala Harter de Autoestima Global.
25^39^	España/2015/Español	Tipo de estudio/Nivel de Evidencia: transversal/IV Muestra: 89 adolescentes con edades entre 12 y 16 años. Lugar: hospital. Instrumento: Perfil de Salud y Enfermedad Infantil.
26^40^	España/2015/Español	Tipo de estudio/Nivel de Evidencia: transversal/VI Muestra: 296 niños con edades entre 12 y 14 años. Lugar: escuela. Instrumento: Escala de Autoestima de Rosenberg.
27^41^	Estados Unidos de América/2014/Inglés	Tipo de estudio/Nivel de Evidencia: casos y controles/IV Muestra: 1419 estudiantes adolescentes. Lugar: escuela. Instrumento: Escala General de Autoestima.
28^42^	Irán/2014/Inglés	Tipo de estudio/Nivel de Evidencia: transversal/VI Muestra: 8.510 adolescentes con edades entre 15 y 17 años. Lugar: escuelas. Instrumento: Cuestionario de Relaciones Multidimensionales Cuerpo-Yo; Inventario de Autoestima de Coopersmith.
29^43^	Estados Unidos de América/2012/Inglés	Tipo de estudio/Nivel de Evidencia: casos y controles/IV Muestra: 101 adolescentes con edades entre 14 y 18. Lugar: escuela. Instrumento: Escala de Autoestima de Rosenberg.
30^44^	Estados Unidos de América/2012/Inglés	Tipo de estudio/Nivel de Evidencia: transversal/IV Muestra: 119 adolescentes con edades entre 11 y 18 años. Lugar: hospital. Instrumento: Inventario de Autoestima de Coopersmith.
31^45^	Estados Unidos de América/2011/Inglés	Tipo de estudio/Nivel de Evidencia: cohorte prospectiva/IV Muestra: 806 adolescentes. Lugar: escuela. Instrumento: Escala de Autoestima de Rosenberg.
32^46^	Estados Unidos de América/2010/Inglés	Tipo de estudio/Nivel de Evidencia: cohorte prospectiva/IV Muestra: 4458 adolescentes con edades entre 12 y 16 años Lugar: remoto. Instrumento: Perfil de Autoestima de Harter para Niños.
33^47^	Suecia/2010/Inglés	Tipo de estudio/Nivel de Evidencia: cohorte prospectiva/IV Muestra: una cohorte de los años 2000 con 191 adolescentes y una cohorte de 2008 con 170 adolescentes. Lugar: escuela. Instrumento: Perfil de Autopercepción Física para Niños y Adolescentes.
34^48^	Francia/2010/Francés	Tipo de estudio/Nivel de Evidencia: ensayo clínico aleatorizado controlado/II Muestra: 16 adolescentes con una media de edad de 14,9 años. Lugar: hospital. Instrumento: Inventario Físico de Sí Mismo.
35^49^	Turquía/2004/Inglés	Tipo de estudio/Nivel de Evidencia: casos y controles/IV Muestra: 90 adolescentes con edades entre 12 y 16 años. Lugar: escuela. Instrumento: Escala de Autoestima de Rosenberg.
36^50^	Reino Unido/2023/Inglés	Tipo de estudio/Nivel de Evidencia: ensayo clínico aleatorizado controlado/II Muestra: 57 adolescentes obesos y 38 adolescentes con peso normal. Lugar: campamento residencial. Instrumento: Perfil de Autopercepción para Niños.
37^51^	Suecia/1999/Inglés	Tipo de estudio/Nivel de Evidencia: casos y controles/IV Muestra: 58 adolescentes con obesidad y 58 con peso normal, con edades entre 14 y 18 años. Lugar: escuela. Instrumento: Cuestionario Yo Pienso, Yo Soy.
38^52^	Francia/1995/Inglés	Tipo de estudio/Nivel de Evidencia: transversal/VI Muestra: 93 adolescentes con edades entre 13 y 19 años. Lugar: hospital. Instrumento: Inventario de Autoestima de Coopersmith.
39^53^	Estados Unidos de América/1988/Inglés	Tipo de estudio/Nivel de Evidencia: transversal/VI Muestra: 550 niñas de 14 y 16 años. Lugar: escuela. Instrumento: Escala de Autoestima de Rosenberg.

*TAE-student = Prueba de Autoestima Escolar

En cuanto al país de origen de los estudios, nueve se realizaron en Estados Unidos de América^23^ ^,^ ^25^ ^,^ ^34^ ^,^ ^41^ ^,^ ^43^ ^-^ ^46^ ^,^ ^53^, cuatro en Chile^17^ ^,^ ^27^ ^-^ ^28^ ^,^ ^31^ y en el Reino Unido^20^ ^-^ ^21^ ^,^ ^37^ ^,^ ^50^, tres en España^29^ ^,^ ^39^ ^-^ ^40^, dos en Brasil^16^ ^,^ ^33^, Francia^48^ ^,^ ^52^, Irán^26^ ^,^ ^42^, Suecia^47^ ^,^ ^51^ y Turquía^19^ ^,^ ^49^, y uno en Arabia Saudita^35^, Australia^11^, Canadá^38^, China^22^, Egipto^30^, India^32^, Italia^36^, Grecia^24^ y Marruecos^18^. Un mapa representativo de los países de origen de las publicaciones se presenta en la Figura 4.

**Figura 4:** País de origen y número de publicaciones correspondientes. Teresina, PI, Brasil, 2024

En cuanto al año de publicación, cuatro se publicaron en 2017^11^ ^,^ ^32^ ^-^ ^34^, tres en 2024^16^ ^-^ ^18^, 2020^24^ ^-^ ^26^, 2018^29^ ^-^ ^31^, 2016^35^ ^-^ ^37^, 2015^38^ ^-^ ^40^ y 2010^46^ ^-^ ^48^, dos en 2023^19^ ^-^ ^20^, 2021^22^ ^-^ ^23^, 2019^27^ ^-^ ^28^, 2014^31^ ^,^ ^42^ y 2012^43^ ^-^ ^44^, uno en 2022^21^, 2011^45^, 2004^49^, 2003^50^, 1999^51^, 1995^52^ y 1988^53^. En cuanto al idioma, la mayoría de los estudios se publicaron en inglés^11^ ^,^ ^16^ ^,^ ^18^ ^-^ ^26^ ^,^ ^28^ ^-^ ^29^ ^,^ ^31^ ^-^ ^32^ ^,^ ^34^ ^,^ ^36^ ^-^ ^38^ ^,^ ^41^ ^-^ ^47^ ^,^ ^49^ ^-^ ^53^, seguido del español^17^ ^,^ ^27^ ^,^ ^20^ ^,^ ^39^ ^-^ ^40^, portugués^33^, árabe^35^ y francés^48^.

Los tipos de estudios desarrollados fueron transversales^17^ ^-^ ^18^ ^,^ ^21^ ^,^ ^23^ ^-^ ^33^ ^,^ ^39^ ^-^ ^40^ ^,^ ^42^ ^,^ ^44^ ^,^ ^52^ ^-^ ^53^, casos y controles^19^ ^,^ ^22^ ^,^ ^36^ ^,^ ^41^ ^,^ ^43^ ^,^ ^50^ ^,^ ^52^, cohortes^20^ ^,^ ^34^ ^,^ ^36^ ^,^ ^46^ ^-^ ^48^, ensayos clínicos aleatorizados controlados^16^ ^,^ ^38^ ^-^ ^39^ ^,^ ^49^ ^,^ ^51^ y una revisión sistemática^11^, con una muestra que varió entre 16^48^ y 18.818^20^ adolescentes. El Nivel de Evidencia se clasificó, de modo que se identificaron estudios de nivel VI^17^ ^-^ ^18^ ^,^ ^21^ ^,^ ^23^ ^-^ ^33^ ^,^ ^39^ ^-^ ^40^ ^,^ ^42^ ^,^ ^44^ ^,^ ^52^ ^-^ ^53^, IV^19^ ^-^ ^20^ ^,^ ^22^ ^,^ ^34^ ^,^ ^36^ ^,^ ^41^ ^,^ ^43^ ^,^ ^46^ ^-^ ^48^ ^,^ ^50^ ^,^ ^52^, II^16^ ^,^ ^38^ ^-^ ^39^ ^,^ ^49^ ^,^ ^51^ e I^11^. Los estudios se realizaron en diferentes escenarios como campamento^50^, centros comunitarios^25^, centro de estudios^26^, centros educativos públicos^29^, escuelas^11^ ^,^ ^17^ ^-^ ^18^ ^,^ ^22^ ^-^ ^24^ ^,^ ^27^ ^-^ ^28^ ^,^ ^30^ ^-^ ^33^ ^,^ ^35^ ^-^ ^36^ ^,^ ^40^ ^-^ ^43^ ^,^ ^45^ ^,^ ^47^ ^,^ ^49^ ^,^ ^51^ ^,^ ^53^, hospitales^33^ ^,^ ^39^ ^,^ ^44^ ^,^ ^48^ ^,^ ^52^, instalaciones comunitarias^38^, remoto^46^, domicilios^11^ ^,^ ^21^ ^,^ ^37^ y universidad^19^, y en uno de ellos no se especificó^20^.

Los instrumentos Autoestima Global de Marsh^11^, Escala de Autoestima de Rosenberg^11^ ^,^ ^16^ ^-^ ^21^ ^,^ ^25^ ^-^ ^26^ ^,^ ^28^ ^,^ ^30^ ^,^ ^32^ ^-^ ^33^ ^,^ ^36^ ^,^ ^40^ ^,^ ^41^ ^,^ ^45^ ^,^ ^49^ ^,^ ^53^ y versión modificada^23^, Escala de Estereotipo Corporal para Adolescentes y Adultos^22^, Escala General de Autoestima^41^, Inventario de Autoestima de Coopersmith^27^ ^,^ ^42^ ^,^ ^44^ ^,^ ^52^, Inventario Físico de Sí Mismo^48^, Perfil de Autoestima de Harter para Niños^46^, Perfil de Autopercepción de Harter para Niños^11^, Perfil de Autopercepción Física para Niños^38^, Perfil de Autopercepción Física para Niños y Adolescentes^47^, Perfil de Autopercepción para Adolescentes^34^, Perfil de Autopercepción para Niños^24^ ^,^ ^37^ ^,^ ^50^, Perfil de Salud y Enfermedad Infantil^39^, Cuestionario Yo Pienso, Yo Soy^51^, Cuestionario de Relaciones Multidimensionales Cuerpo-Yo^42^, Subescala Harter de Autoestima Global^38^ y *TAE-student*: prueba de autoestima escolar^29^ ^-^ ^31^ se utilizaron para evaluar la autoestima entre los participantes.

Los estudios identificaron que la obesidad y el aumento del Índice de Masa Corporal (IMC) influyen en la baja autoestima entre adolescentes, destacando esta prevalencia en el sexo femenino^11^ ^,^ ^17^ ^-^ ^24^ ^,^ ^27^ ^-^ ^30^ ^,^ ^33^ ^,^ ^39^ ^-^ ^43^ ^,^ ^45^ ^-^ ^47^ ^,^ ^51^ ^-^ ^53^. Se señala que la baja autoestima contribuye a la vergüenza corporal^22^ ^,^ ^36^, uno de los predictores de trastornos alimentarios, así como está influenciada por la exposición a la victimización por pares^41^ ^,^ ^44^, se refleja en impactos negativos en la salud mental y el bienestar^19^ ^-^ ^21^ ^,^ ^26^ ^,^ ^32^ ^,^ ^41^ ^,^ ^43^ ^,^ ^48^ ^-^ ^49^ y en el rendimiento escolar^27^ ^,^ ^34^ ^,^ ^35^. Se destaca la necesidad de intervenciones para la promoción de la autoestima en este público, como la práctica deportiva y de actividad física, y la importancia del apoyo familiar^16^ ^,^ ^25^ ^-^ ^26^ ^,^ ^37^ ^-^ ^38^ ^,^ ^40^ ^,^ ^50^. Los objetivos y principales resultados de los estudios se describen en la Figura 5.

**Figura 5:** Descripción de los estudios seleccionados en cuanto a los objetivos y principales resultados. Teresina, PI, Brasil, 2024

Orden	Objetivos	Principales resultados
01^16^	Analizar los efectos de 16 semanas de práctica de Muay Thai en la percepción corporal, la autoestima y la CV* en adolescentes con sobrepeso/obesidad.	Se observó un aumento en los niveles de autoestima entre los participantes del grupo que realizó prácticas de Muay Thai.
02^17^	Relacionar la insatisfacción corporal con autoestima, depresión e IMC^†^ en adolescentes.	De la muestra, el 17,1% eran obesos y el 1,5% eran gravemente obesos. Se observó la prevalencia de baja autoestima en el sexo femenino. Se encontraron correlaciones entre las variables de insatisfacción corporal y el puntaje z del IMC^†^, síntomas depresivos y autoestima.
03^18^	Evaluar los niveles de autoestima, depresión y su asociación con obesidad/exceso de peso.	En la categoría de obesidad, las niñas presentaron una autoestima significativamente más baja en comparación con los niños. Se encontró una fuerte asociación entre depresión y obesidad/exceso de peso, y baja autoestima.
04^19^	Investigar los efectos de la obesidad en la ansiedad, la depresión, la autoestima y la regulación emocional en niños y adolescentes	Los análisis secundarios mostraron que los niños con obesidad tenían una autoestima más baja que aquellos con síntomas de ansiedad y depresión. También se encontró correlación entre síntomas de depresión, ansiedad y dificultades en la regulación emocional en el grupo con obesidad.
05^20^	Determinar las trayectorias de intervención entre la salud mental y los síntomas del puntaje z del IMC^†^ a lo largo de la adolescencia.	Se encontraron evidencias de que la relación entre IMC^†^ y salud mental fue parcialmente atribuible a la felicidad con la apariencia y la autoestima. Para niños y niñas, síntomas emocionales elevados en comparación con síntomas bajos/moderados y aumento en el puntaje z del IMC^†^ a los 11 años se asociaron con mayores probabilidades de infelicidad con la apariencia y baja autoestima a los 14 años.
06^21^	Investigar características específicas de género asociadas con la baja participación deportiva entre adolescentes del Reino Unido y evaluar el seguimiento del IMC^†^ específico de género y las asociaciones específicas de género entre IMC^†^ y autoestima con base en diferentes niveles de participación deportiva de adolescentes.	Niños y niñas en el cuartil más bajo de participación deportiva tenían mayor probabilidad de presentar sobrepeso/obesidad y baja autoestima en comparación con adolescentes en el cuartil más alto de participación deportiva.
07^22^	Explorar cómo la Inteligencia Emocional, el género y el tamaño corporal influyen en la relación entre la autoestima corporal y el riesgo de trastornos alimentarios entre adolescentes.	El género y el tamaño corporal influyeron directamente en la autoestima corporal y en el riesgo de trastornos alimentarios. Además, la Inteligencia Emocional actuó como moderadora entre la autoestima corporal y el riesgo de trastornos.
08^23^	Ampliar la literatura al utilizar una medida relativa del peso corporal o la clasificación del IMC^†^ de un alumno dentro de su grupo de pares, relevante para examinar el impacto del peso corporal en los resultados de los jóvenes.	En estudiantes del sexo femenino con IMC^†^ alto existe probabilidad de experimentar una disminución de la autoestima y un aumento de los síntomas depresivos.
09^24^	Investigar el exceso de peso corporal y la obesidad abdominal en relación con la autopercepción, la autoestima y la ansiedad.	Los participantes con sobrepeso y obesidad presentaron puntajes más bajos en todos los dominios de la autoestima global en comparación con niños con peso normal.
10^25^	Examinar el papel de factores culturales (por ejemplo, familismo) y psicosociales (por ejemplo, autoestima) como predictores de la CV* específica del peso entre adolescentes latinos con obesidad.	El familismo y la autoestima positiva pueden servir como factores de protección asociados con una mayor CV* en adolescentes con obesidad, mientras que la autodepreciación puede ser un factor de riesgo para una menor CV* asociada al peso.
11^26^	Investigar el impacto de la comunicación y el funcionamiento familiar, y la satisfacción con la imagen corporal, en el bienestar psicológico considerando: el papel mediador de la autoestima y la depresión.	Los resultados mostraron que la comunicación y el funcionamiento familiar afectaron directamente el bienestar psicológico de los adolescentes. Además, la comunicación y el funcionamiento familiar, así como la satisfacción con la imagen corporal, afectaron indirectamente el bienestar psicológico por medio de la autoestima y la depresión.
12^27^	Determinar la asociación entre los niveles nutricionales, la aptitud física, la autoestima y la CV* con el rendimiento académico en adolescentes.	Los alumnos con obesidad presentaron menor CVRS^‡^ que sus pares con sobrepeso y peso normal. Los escolares con sobrepeso y obesidad presentaron niveles más bajos de autoestima, lo cual se relacionó con un bajo rendimiento académico.
13^28^	Medir la prevalencia de sobrepeso y obesidad e identificar su relación con la autoestima entre alumnos adolescentes.	Los resultados del estudio muestran que 71 (13,3%) adolescentes son obesos. Además, se identificó una asociación estadísticamente significativa entre autoestima e IMC^†^, en la que la baja autoestima estuvo presente en cerca de tres cuartos de los adolescentes con sobrepeso y obesidad.
14^29^	Determinar si existe asociación entre la insatisfacción con la imagen corporal y parámetros antropométricos, estado de peso y autoestima en niños de escuelas públicas.	La insatisfacción con la imagen corporal fue mayor entre niñas y niños con obesidad, quienes también presentaron menor autoestima.
15^30^	Relacionar la condición de sobrepeso y obesidad con los niveles de autoestima y felicidad en escolares de centros educativos públicos.	La autoestima escolar fue significativamente mayor en los niños. El exceso de peso se asoció con la infelicidad en niños en edad escolar.
16^31^	Evaluar la percepción de escolares obesos respecto de su participación en las clases de educación física y determinar su autoestima en comparación con los grupos con sobrepeso y peso normal.	Los estudiantes con obesidad se sienten excluidos de las clases de educación física y presentan bajos niveles de autoestima, en comparación con escolares con peso normal.
17^32^	Examinar si las adolescentes con obesidad/sobrepeso difieren de las adolescentes con peso normal en términos de autoestima.	El análisis correlacional de los datos reveló una relación negativa del IMC^†^ con la autoestima. Los hallazgos sugirieron que existe necesidad de mejorar la autoestima de las adolescentes, que generalmente disminuye debido a la percepción del peso corporal, así como por los factores sociales y psicológicos involucrados.
18^33^	Investigar la autoestima, la imagen corporal y la depresión de adolescentes en diferentes estados nutricionales.	Los niños presentaron mayor prevalencia de sobrepeso y obesidad, menor depresión e insatisfacción corporal y mayor autoestima que las niñas. La depresión tuvo relación negativa con la autoestima, que también se asoció negativamente con la insatisfacción corporal.
19^34^	Examinar las asociaciones de la victimización entre pares con síntomas internalizantes, competencia social y rendimiento académico en una muestra clínica de adolescentes con obesidad grave y si la autoestima y el apoyo social afectan estas asociaciones.	La autoestima medió los efectos de la victimización, siendo importante para la prevención e intervención tanto de la victimización como del desajuste en la obesidad grave entre adolescentes.
20^11^	Investigar el impacto de intervenciones multicomponente de control de peso en la autoestima en adolescentes con sobrepeso y obesidad.	Los adolescentes con sobrepeso y obesidad tienden a tener baja autoestima. El metanálisis no encontró una mejora significativa en la autoestima, incluso con pérdida de peso. Sin embargo, las intervenciones con mayores efectos en la autoestima también presentaron mayor efecto sobre la pérdida de peso.
21^35^	Evaluar la asociación entre sobrepeso/obesidad y el rendimiento académico general futuro entre estudiantes de enseñanza media en Arabia Saudita.	En un modelo de regresión logística múltiple ajustado por características sociodemográficas, estilo de vida relacionado con el estudio y autoestima, el sobrepeso/obesidad se asoció con el declive del rendimiento académico.
22^36^	Investigar comportamientos alimentarios disfuncionales y variables psicológicas típicamente asociadas a trastornos alimentarios, como baja autoestima, perfeccionismo, vergüenza, cuidado parental percibido y sobreprotección en adolescentes obesos y con peso normal, y examinar cómo los principales factores de riesgo para trastornos alimentarios interactúan entre sí, lo que explica la vulnerabilidad a la psicopatología alimentaria.	La autoestima no tuvo efecto directo en el riesgo de trastorno alimentario. La vergüenza corporal tuvo una relación más fuerte con la vulnerabilidad a problemas alimentarios; sin embargo, la baja autoestima tuvo una influencia directa en la vergüenza corporal que, a su vez, afecta el riesgo de psicopatología alimentaria.
23^37^	Evaluar la autocompetencia percibida y la baja autoestima durante un programa intensivo de pérdida de peso en una gran muestra de jóvenes obesos y relacionarlas con el peso inicial, el género y la pérdida de peso.	Los participantes que asistieron al campamento de verano obtuvieron mejoras en la autoestima global, reduciendo la baja autoestima del 35% al 16%.
24^38^	Determinar los efectos del entrenamiento aeróbico, el entrenamiento de resistencia y el entrenamiento combinado en el estado de ánimo, la imagen corporal y la autoestima en adolescentes con obesidad.	A partir de las intervenciones de entrenamiento aeróbico, de resistencia y combinado, se registró que todos los grupos mejoraron la imagen corporal y las autopercepciones físicas. El entrenamiento de resistencia mostró mayores aumentos en la autoestima global.
25^39^	Evaluar la influencia del estado ponderal en la CVRS^‡^ de los adolescentes.	Los adolescentes con sobrepeso/obesidad presentaron peor CVRS^‡^, menor resistencia, menor capacidad para la actividad física, menor participación familiar y mayor influencia de los pares. La autoestima fue menor entre las participantes del sexo femenino.
26^40^	Analizar la influencia de la composición corporal sobre la autoestima en niños de 12 a 14 años en la ciudad de Jaén, España.	Se observaron valores estadísticamente menores de autoestima en las niñas en comparación con los niños. Un IMC^†^ más alto también se asoció con baja autoestima.
27^41^	Examinar diferencias de grupo relacionadas con burlas en aspectos de bienestar psicológico, autopercepciones y comportamientos.	Se identificó que existe relación entre burlas sobre el peso y menor bienestar psicológico. Los adolescentes que informaron sufrir burlas presentaron menor autoestima, más síntomas depresivos y se percibían como menos capaces físicamente en comparación con quienes no fueron objeto de burlas.
28^42^	Examinar la satisfacción de los adolescentes con su imagen corporal correlacionada con su autoestima.	La autoestima fue baja entre los adolescentes con obesidad. La menor satisfacción con la imagen corporal conduce a la baja autoestima.
29^43^	Comparar los niveles de estrés, autoestima, afrontamiento, apoyo social y estado de ánimo depresivo entre adolescentes de minorías con peso normal y con sobrepeso/obesidad.	Los adolescentes con obesidad/sobrepeso informaron una autoestima significativamente menor que aquellos con peso normal. La baja autoestima también fue un predictor significativo del estado de ánimo depresivo en el grupo con sobrepeso/obesidad.
30^44^	Examinar correlatos basados en el peso, autoestima y depresión entre 119 adolescentes obesos afroamericanos de 11 a 18 años en busca de tratamiento.	Los resultados indicaron una asociación entre burlas y disminución de la autoestima y aumento de la depresión. La satisfacción corporal medió parcialmente la asociación entre burlas y autoestima.
31^45^	Probar la hipótesis de que la insatisfacción corporal media la asociación entre obesidad y deterioro del bienestar emocional en dos momentos de la vida de una muestra de la población general de adolescentes del sexo masculino y femenino.	Los participantes obesos tienden a tener menor autoestima y mayor estado de ánimo depresivo que los participantes con peso normal. Además, los niveles absolutos de insatisfacción corporal y estado de ánimo depresivo permanecieron más altos y los niveles de autoestima más bajos en las niñas, independientemente del peso corporal.
32^46^	Examinar características asociadas a la baja autoestima en una gran muestra nacional de adolescentes jóvenes.	En el análisis multivariado, sexo femenino, raza hispánica, sobrepeso y obesidad, búsqueda de sensaciones, rebeldía y tiempo diario de televisión se asociaron de manera independiente con baja autoestima.
33^47^	Explorar tendencias seculares entre 2000 y 2008 en la autoestima física comparando cohortes transversales de adolescentes jóvenes.	Hubo una Autoestima Global significativamente mayor en la cohorte de 2008 en comparación con la de 2000. En la cohorte de 2008, en el grupo de adolescentes con sobrepeso/obesidad, la única diferencia significativa fue una Fuerza Física percibida menor en las niñas y una Autoestima Global significativamente menor en los niños.
34^48^	Analizar las relaciones entre los valores de ansiedad, autoestima global y percepción del esfuerzo durante ejercicio físico de intensidad creciente en una población de adolescentes obesas y no obesas.	Los valores de ansiedad registrados antes del ejercicio se correlacionaron significativamente con los valores globales de autoestima en el grupo con obesidad.
35^49^	Explorar el tipo y la frecuencia de la psicopatología en una muestra clínica y no clínica de adolescentes obesos y en un grupo control con peso normal.	Hubo una mayor proporción de psicopatología (depresión, problemas conductuales, baja autoestima) entre adolescentes obesos clínicos que entre adolescentes obesos no clínicos. Los hallazgos aportaron evidencias de una población de riesgo psicosocial en un subgrupo de adolescentes con obesidad.
36^50^	Investigar el cambio en la imagen corporal, autoestima y preocupaciones en adolescentes obesos que asisten a un campamento residencial para pérdida de peso.	Los participantes presentaban mayor insatisfacción con el peso corporal y con la autoestima. La participación en el campamento de pérdida de peso mejoró el estado psicológico de los adolescentes y los niveles globales de autoestima.
37^51^	Comparar la autoestima, el historial social, la competencia social y académica, problemas conductuales y estilo de vida de adolescentes con obesidad.	La obesidad tuvo una correlación no significativa con la autoestima autoevaluada. Aunque esta relación fue limitada, los participantes obesos demostraron insatisfacción con su apariencia física y habilidades atléticas, como era de esperar. Estos hallazgos indican que, a pesar del exceso de peso, la percepción de la autoestima de los adolescentes no fue fuertemente influenciada por la obesidad.
38^52^	Estudiar trastornos alimentarios y emocionales en niñas adolescentes con Diabetes Mellitus insulinodependiente.	Las niñas con obesidad y con o sin Diabetes Mellitus presentaron menores puntuaciones en el puntaje total de autoestima.
39^53^	Investigar la autoestima de niñas adolescentes en relación con el peso.	Los resultados demuestran que la autoestima de las niñas adolescentes está relacionada con su peso. A medida que aumentó el índice de obesidad, la autoestima disminuyó.

*CV = Calidad de Vida; ^†^IMC = Índice de Masa Corporal; ^‡^CVRS = Calidad de Vida Relacionada con la Salud

## Discusión

El mapeo de los estudios permitió identificar cómo se caracteriza la autoestima en adolescentes con obesidad, sus impactos y posibles intervenciones para su mejora. La mayoría de los estudios señala que el diagnóstico de obesidad en adolescentes se relaciona con una menor autoestima^11^ ^,^ ^17^ ^-^ ^19^ ^,^ ^21^ ^,^ ^24^ ^,^ ^27^ ^,^ ^31^ ^,^ ^41^ ^-^ ^43^ ^,^ ^51^. La relación entre la autoestima y el IMC fue examinada en diferentes investigaciones que revelaron una correlación negativa y significativa, lo que sugiere que, a medida que aumenta el IMC, disminuye la autoestima^20^ ^,^ ^30^ ^,^ ^32^. También puede observarse una relación inversa en la literatura, lo que indica que presentar baja autoestima es uno de los principales factores que contribuyen a la obesidad y afecta su tratamiento^19^ ^,^ ^54^.

La obesidad se refleja en una menor autoestima, pues puede influir de manera negativa en la autopercepción durante la adolescencia, principalmente en el sexo femenino^18^ ^,^ ^22^ ^-^ ^23^ ^,^ ^28^ ^-^ ^29^ ^,^ ^33^ ^,^ ^39^ ^-^ ^40^ ^,^ ^45^ ^-^ ^46^ ^,^ ^52^ ^-^ ^53^. Esta diferencia entre géneros está influida por factores socioculturales, familiares y experiencias individuales. Los medios de comunicación, los estándares de belleza considerados aceptables, las expectativas sociales y las presiones académicas pueden impactar la autoestima de forma distinta, lo que contribuye a la formación de identidades y autopercepciones únicas entre los sexos^55^.

La baja autoestima se asocia con insatisfacción con la vida, problemas familiares, menor apoyo social percibido, salud emocional y bienestar negativos y trastornos mentales, como depresión y ansiedad, y se considera un predictor de mayores tasas de suicidio en este grupo^23^ ^,^ ^32^ ^,^ ^41^ ^,^ ^43^ ^,^ ^49^ ^,^ ^56^ ^-^ ^57^. Otro aspecto es que los síntomas de ansiedad y depresión, comunes en la obesidad, contribuyen a la disminución de los niveles de autoestima^19^ ^-^ ^20^ ^,^ ^45^. Se destaca que la influencia de la imagen corporal, la aceptación social y el deterioro del estado de ánimo es mayor en el sexo femenino, lo que lo hace más vulnerable a los trastornos psicosociales^58^ ^-^ ^59^.

La baja autoestima entre adolescentes con obesidad también puede considerarse un factor de riesgo para manifestaciones clínicas de trastornos alimentarios, principalmente en mujeres, ya que la búsqueda del cuerpo considerado “ideal” puede llevar a la adopción de comportamientos alimentarios inadecuados^22^. La baja autoestima también influye directamente en la vergüenza corporal, que, a su vez, es un predictor de riesgo para psicopatologías alimentarias^36^.

El prejuicio contra la obesidad y las burlas respecto al peso son comunes en el medio social^41^. Hay evidencias de que ser obeso se identifica como una de las condiciones menos aceptables socialmente y posiblemente más estigmatizantes, principalmente en la infancia y la adolescencia^60^. Los niveles de autoestima de niños con obesidad disminuyen con la edad y las dificultades psicosociales se vuelven más pronunciadas a medida que crecen y entran en la adolescencia, pues los adolescentes con obesidad son más propensos a sufrir burlas de sus pares, lo que puede conducir a la marginación social^24^ ^,^ ^61^.

La exposición a burlas relacionadas con el peso, la intimidación o la victimización también contribuye al desarrollo de problemas psicológicos y emocionales, que llevan a un bajo rendimiento escolar^27^ ^,^ ^34^ ^-^ ^35^. También se informó que el rendimiento escolar está influido por el exceso de peso o por el absentismo de estudiantes con obesidad debido a los problemas de salud que pueden enfrentar^24^. Se han sugerido diferentes explicaciones para la asociación negativa entre IMC y rendimiento académico, y una de ellas incluye el impacto negativo en la autoestima^35^.

Aunque esta revisión destaca la relación negativa entre obesidad y autoestima, esta última, cuando se evalúa positivamente, se relaciona con mejores condiciones de bienestar y salud mental, autopercepciones positivas, logros académicos y persistencia^31^. Esto refuerza la importancia de la prevención y el control de la obesidad aún en la infancia, así como estrategias de asesoramiento para ayudar en el control del exceso de peso en adolescentes^46^.

Los estudios demuestran que los adolescentes con mejor rendimiento escolar y con participación en deportes colectivos presentan menor riesgo de baja autoestima, lo que destaca una oportunidad para incentivar el desarrollo de programas deportivos escolares y comunitarios que colaboren con la prevención de la obesidad y la promoción de una mejor autoestima^21^ ^,^ ^31^ ^,^ ^46^. Los individuos con mayor autoestima también se sienten más motivados para practicar actividad física^18^ ^,^ ^21^ ^,^ ^62^.

Las prácticas deportivas, como el Muay Thai, se aplicaron como intervención para mejorar la autoestima entre adolescentes con obesidad^16^. Estas prácticas aportan contribuciones no solo a la salud física y psíquica, sino que también favorecen las competencias sociales^63^. Un estudio que ofreció un programa de Kárate con duración de más de diez semanas para niños y adolescentes con epilepsia mostró mejoras en la autoestima de los practicantes^64^. Otro analizó la autoestima, la autoconfianza y la ansiedad cognitiva y somática en adultos practicantes y no practicantes de Jiu-Jitsu, y observó que los practicantes presentaron mayores niveles de autoestima y autoconfianza^65^.

Los entrenamientos aeróbicos y de resistencia, así como la combinación de ambos, demostraron efectos positivos en la salud psicológica de adolescentes con obesidad. Se verificó que la práctica, con una frecuencia de al menos seis meses, resultó en mejoras en la autoestima global y física. Los factores asociados al efecto del entrenamiento sugieren el potencial de remodelación corporal y mejoras en el tamaño corporal, la forma y el tono muscular, además de la reducción del IMC^38^.

Las intervenciones realizadas en campamentos presentaron potenciales efectos en la autoestima global de los adolescentes; esto se debe a la combinación de actividades desarrolladas, con énfasis en los aspectos nutricionales, físicos y emocionales. Un predictor asociado a mejoras en el nivel de autoestima también fue la reducción del peso. En uno de los estudios que utilizó la intervención en un entorno de campamento, los participantes tuvieron en promedio una reducción del peso de 5,6 kg^50^.

Además, las intervenciones con ambientación de campamento también obtuvieron mejoras significativas en la autoestima global de los adolescentes con obesidad, incluso cuando no había una reducción significativa de peso aparente, lo que puede estar asociado con la autoeficacia, como la práctica de ejercicio regular y un entorno de apoyo que pueden favorecer el desarrollo de competencias y mayores interacciones sociales, estableciendo nuevas amistades o estando en un ambiente menos vulnerable a experiencias asociadas al estigma y la victimización^37^.

Las intervenciones de carácter educativo presentan múltiples beneficios; esto ocurre debido a la naturaleza de estas intervenciones, de explorar diferentes habilidades como el aprendizaje, los comportamientos y las actitudes, posibilitando modificaciones y reflexiones acerca de la autoestima, alejándolos de los estigmas, las burlas de los compañeros y los patrones estéticos socialmente definidos, acercándolos a creencias y valores congruentes con la autovaloración y la autoconfianza en sí mismos. Cabe resaltar que el período de la intervención es fundamental para garantizar los efectos potenciales. La aplicación de un programa educativo durante 12 meses fue capaz de identificar mejoras en la satisfacción corporal, cambios de actitudes y percepción relacionados con la autoestima^40^.

Se refuerza también la importancia de las intervenciones basadas en el familismo, en las cuales, en un estudio realizado con adolescentes latinos con obesidad, la relación familiar representó un factor de protección para la autoestima y, en consecuencia, se asoció con el bienestar general. Las familias estructuradas y estables pueden proporcionar al adolescente una visión más amplia sobre sus cualidades y valores que van más allá del peso y la apariencia física, brindando una red de apoyo y competencias para desarrollar relaciones interpersonales con mayor facilidad y participar en actividades alineadas con sus objetivos^25^.

El período de la infancia desempeña un papel importante en la formación de la autoestima de niños y adolescentes. Los cuidadores sensibles y solidarios contribuyen a la formación del apego en los niños. A medida que reciben retroalimentación positiva, forman un autoconcepto positivo, lo que los hace más seguros y con mejor autoestima^26^. La autoestima formada durante la infancia puede continuar durante la adolescencia. Así, la familia es uno de los factores más importantes que influyen en la autoestima de los adolescentes, especialmente entre las niñas^66^.

Una revisión sistemática identificada señaló que las intervenciones multicomponente, tales como alimentación, educación y actividad física, demostraron beneficios en la mejora de la autoestima, a pesar de que la relación entre autoestima y obesidad no está totalmente esclarecida. La adopción de estas estrategias, con foco en la autoestima del adolescente, puede ser eficiente para la reducción del IMC y la mejora en la calidad de vida de estos individuos. En cuanto a los instrumentos utilizados para evaluar la autoestima, se identificaron dos tipos diferentes de instrumentos, entre ellos: la Escala de Autoestima de Rosenberg y el Perfil de Autopercepción de Harter para Niños/Adolescentes^11^.

Estos datos corroboran los encontrados en esta revisión, en la que la evaluación de la autoestima se realizó de forma cuantitativa, apoyándose en el uso de instrumentos validados, siendo el más utilizado la Escala de Autoestima de Rosenberg^11^ ^,^ ^16^ ^-^ ^21^ ^,^ ^25^ ^-^ ^26^ ^,^ ^30^ ^,^ ^32^ ^-^ ^34^ ^,^ ^40^ ^-^ ^41^ ^,^ ^45^ ^,^ ^49^ ^,^ ^53^. También se utilizaron las versiones validadas y adaptadas a los idiomas portugués^33^ y español^17^. A continuación, se encuentra el Inventario de Autoestima de Coopersmith, que evalúa la autoestima en diferentes áreas: familiar, académica, social y general^27^ ^,^ ^41^ ^,^ ^44^ ^,^ ^52^; y la escala Perfil de Autopercepción de Harter para Niños/Adolescentes, que evalúa de manera más detallada los aspectos de la vida de los individuos en cuanto a la dimensión global de la autoestima en diferentes dimensiones o dominios de la vida^11^ ^,^ ^38^.

La variedad de instrumentos identificados en la síntesis de resultados resalta la complejidad de la evaluación de la autoestima, así como la investigación de este concepto en diferentes aspectos del individuo, ya sea en enfoques más abarcadores como la autoestima global o en componentes específicos dentro de la autoestima y cómo estos se relacionan e impactan a nivel psicológico y social en la vida de las personas, incluidos los adolescentes.

Los estudios transversales se utilizaron ampliamente para evaluar la autoestima entre los adolescentes y correlacionarla con otras variables^17^ ^-^ ^18^ ^,^ ^21^ ^,^ ^23^ ^-^ ^33^ ^,^ ^39^ ^-^ ^40^ ^,^ ^42^ ^,^ ^44^ ^,^ ^52^ ^-^ ^53^. Este tipo de estudio es útil para determinar la prevalencia de una condición de interés en una población en un determinado período de tiempo, identificar asociaciones, generar hipótesis para investigaciones futuras y contribuir a la planificación de intervenciones. Sin embargo, presenta limitaciones, como no permitir establecer relaciones de causalidad ni analizar la evolución de un determinado constructo a lo largo del tiempo. Además, es susceptible a sesgos de selección, ya que la muestra de la población de interés puede no ser representativa, comprometiendo la generalización de los resultados^67^.

Además, el papel de los equipos de salud es fundamental para el cribado y la identificación precoz de adolescentes con altos niveles de estrés y baja autoestima. Para ello, estos profesionales pueden actuar mediante programas de promoción de la salud escolar, considerando que este es un entorno de acceso a los adolescentes, pudiendo utilizar instrumentos confiables y validados, exámenes de salud mental, además de proyectos de asesoramiento e integración escolar^23^ ^,^ ^43^.

En este punto, se destaca que la multidisciplinariedad del equipo es algo esencial para la construcción de una red de apoyo a los adolescentes con obesidad, en la que cada profesional desempeña un papel clave. El equipo puede estar constituido por padres, docentes, trabajadores sociales, orientadores y psicólogos, estableciendo una fuerza de tarea capaz de promover un entorno de apoyo que permita a los adolescentes desarrollar habilidades, competencias y confianza para abordar cuestiones de salud mental, obesidad y autoestima^22^ ^-^ ^23^ ^,^ ^43^ ^,^ ^45^.

Cabe señalar que los enfermeros se encuentran en una posición favorable, pues, en lo que respecta a la promoción de la salud escolar, es este profesional quien establece una comunicación previa con docentes, padres, alumnos y otros miembros del personal. Al observar y conversar con los adolescentes, es posible identificar signos de cansancio, estado de ánimo depresivo y desánimo, que pueden alertar a los enfermeros sobre la necesidad de derivar a este adolescente para una evaluación más completa con otros profesionales de salud mental^36^ ^,^ ^41^ ^,^ ^43^.

Otro punto importante es que los profesionales de la salud deben ser conscientes de los efectos de la baja autoestima en la salud mental de los adolescentes con obesidad, considerando que las intervenciones precoces y otros abordajes terapéuticos generalmente se centran en el peso corporal; sin embargo, también deben estar alineados con la autoestima, que influye tanto en el bienestar del adolescente como en la presencia de sufrimiento psíquico^23^ ^,^ ^45^.

Como limitación de esta revisión, se destaca el enfoque utilizado por los estudios incluidos. A pesar del uso de instrumentos validados para medir la autoestima entre adolescentes con obesidad, es importante señalar que la autoestima es un concepto complejo, influido por diferentes variables y contextos, los cuales deben considerarse para una evaluación más abarcadora. Otra limitación se refiere al hecho de que, en algunos estudios, la autoestima se evaluó solo en el momento de la aplicación de la intervención, siendo necesario realizar evaluaciones posteriores a la intervención, ya que la autoestima puede variar a lo largo del tiempo y verse afectada por múltiples factores.

Los hallazgos de este estudio contribuyen a avances en el área de la Enfermería al profundizar la comprensión sobre la autoestima de adolescentes con obesidad. Además, la identificación de los factores asociados con la baja autoestima y las vulnerabilidades presentes en este grupo, principalmente la influencia de los patrones sociales impuestos al sexo femenino, requiere preparación y una mirada atenta de este profesional. Asimismo, se destaca el papel del Enfermero como educador en salud al orientar potenciales intervenciones para la promoción de la autoestima, considerando que las mejoras en este aspecto impactan directamente en el bienestar. El estudio aporta evidencias para la práctica clínica y para estrategias de cuidado holístico y multiprofesional más eficaces y sensibles al contexto psicosocial del adolescente.

## Conclusión

La adolescencia es un período de cambios en el que ocurren oscilaciones psicológicas, ya sea vinculadas al estado de ánimo, a las emociones o a la autoestima. Cuando se asocian con problemas de salud, estas alteraciones pueden ocurrir de forma más intensa, lo que resulta en impactos negativos sobre la salud mental.

Los resultados de algunos estudios incluidos en la revisión indican que la obesidad en adolescentes favorece la baja autoestima y que, en algunos casos, se consideró un predictor del aumento del IMC. Ante ello, se destaca que la baja autoestima se identifica con frecuencia en el público femenino, factor que puede relacionarse con las presiones sociales y las influencias de los pares y de los medios respecto de los cánones de belleza impuestos y aceptados por la sociedad.

La baja autoestima afecta diferentes dimensiones de la vida del adolescente con obesidad, tales como la física, la mental y la social. Además, amplía las vulnerabilidades a trastornos mentales y alimentarios, contribuyendo, en casos más graves, al riesgo de conductas suicidas.

Se destaca que las intervenciones a nivel escolar y en la vida cotidiana de los adolescentes, basadas en la reeducación de hábitos sedentarios, la actividad física y las prácticas deportivas, así como en el énfasis en los aspectos emocionales y en el desarrollo de competencias y habilidades, se presentan como potenciales para la mejora de la autoestima. El papel de la familia es esencial, al ofrecer apoyo y servir como ejemplo de valoración de la singularidad del adolescente, percibiéndolo más allá del diagnóstico de obesidad, considerando sus necesidades, gustos, cualidades y expectativas.

Se sugiere la realización de nuevos estudios, principalmente de carácter cualitativo, que exploren con mayor profundidad y complejidad la percepción de adolescentes con obesidad en relación con la autoestima, considerando las dimensiones biopsicosociales de este público. La inversión en investigaciones en el área favorece el desarrollo de estrategias de intervención orientadas a la promoción de la salud y del bienestar de los adolescentes. 

## Data Availability

Todos los datos generados o analizados durante este estudio están incluidos en este artículo publicado.
